# APOBEC3A cytidine deaminase induces RNA editing in monocytes and macrophages

**DOI:** 10.1038/ncomms7881

**Published:** 2015-04-21

**Authors:** Shraddha Sharma, Santosh K. Patnaik, R. Thomas Taggart, Eric D. Kannisto, Sally M. Enriquez, Paul Gollnick, Bora E. Baysal

**Affiliations:** 1 Department of Pathology, Roswell Park Cancer Institute, Elm and Carlton Streets, Buffalo, New York 14203, USA; 2 Department of Thoracic Surgery, Roswell Park Cancer Institute, Elm and Carlton Streets, Buffalo, New York 14203, USA; 3 Department of Biological Sciences, University at Buffalo, State University of New York, Buffalo, New York 14260, USA

## Abstract

The extent, regulation and enzymatic basis of RNA editing by cytidine deamination are incompletely understood. Here we show that transcripts of hundreds of genes undergo site-specific C>U RNA editing in macrophages during M1 polarization and in monocytes in response to hypoxia and interferons. This editing alters the amino acid sequences for scores of proteins, including many that are involved in pathogenesis of viral diseases. APOBEC3A, which is known to deaminate cytidines of single-stranded DNA and to inhibit viruses and retrotransposons, mediates this RNA editing. Amino acid residues of APOBEC3A that are known to be required for its DNA deamination and anti-retrotransposition activities were also found to affect its RNA deamination activity. Our study demonstrates the cellular RNA editing activity of a member of the APOBEC3 family of innate restriction factors and expands the understanding of C>U RNA editing in mammals.

## Abstract (web summary)

Aberrant RNA editing is linked to a range of neuropsychiatric and chronic diseases. Here Sharma *et al*. show that APOBEC3A can function as an RNA editing protein in response to physiological stimuli, significantly expanding our understanding of RNA editing and the role this may play in diseases.

## Graphical abstract

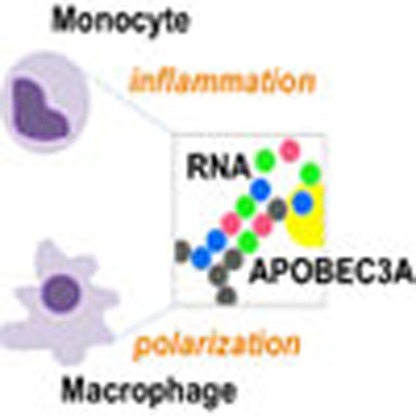

RNA editing is a co- or posttranscriptional process that alters transcript sequences without any change in the encoding DNA sequence^1^. Although various types of RNA editing have been observed in single-cell organisms to mammals, base modifications by deamination of adenine to inosine (A>I) or cytidine to uracil (C>U) are the major types of RNA editing in higher eukaryotes. I and U are read as guanosine (G) and thymine (T), respectively, by the cellular machinery during messenger RNA translation and reverse transcription. RNA editing can therefore alter amino acid sequences, thereby modifying and diversifying protein functions. Aberrant RNA editing is linked to neuropsychiatric diseases such as epilepsy and schizophrenia, and chronic diseases such as cancer^1^.

RNA-dependent ADAR1, ADAR2 and ADAR3 adenosine deaminases, and APOBEC1 cytidine deaminase (CDA) are the only known RNA-editing enzymes in mammals. RNA sequencing studies suggest that A>I RNA editing affects hundreds of thousands of sites, although most of A>I RNA edits occur at a low level and in non-coding intronic and untranslated regions, especially in the context of specific sequences such as Alu elements^2^,^3^,^4^. A>I editing of protein-coding RNA sequences at a high level (>20%) is rare and thought to occur predominantly in the brain. Unlike A>I editing catalysed by adenosine deaminases^5^, the prevalence and level of C>U RNA editing in different types of cells and its enzymatic basis and regulation are poorly understood. The activation-induced deaminase (AID), apolipoprotein B-editing catalytic polypeptide-like (APOBEC) family and CDA proteins of mammals harbour the CDA motif for hydrolytic deamination of C to U^6^. The CDA enzyme is involved in the pyrimidine salvaging pathway. Although AID causes C>U deamination of DNA, multiple studies have failed to identify any RNA-editing activity for this protein^7^. Humans have ten *APOBEC* genes (*APOBEC1*, *2*, *3A–D*, *3F–H* and *4*). APOBEC3 proteins can deaminate cytidines in single-stranded (ss) DNA, and although the APOBEC proteins bind RNA^8^ C>U deamination of RNA is known for only APOBEC1, with apolipoprotein B (*APOB)* mRNA as its physiological target^9^. C>U RNA editing alters hundreds of cytidines in chloroplasts and mitochondria of flowering plants, but the underlying deaminating enzymes are unknown^10^.

We have previously observed C>U editing of cytidine at c.136 (NCBI reference sequence NM_003000), which generates a nonsense codon (R46X), in ∼6% of transcripts of the succinate dehydrogenase B (*SDHB*) gene in normal peripheral blood mononuclear cells (PBMCs) of humans^11^. *SDHB* encodes the iron-sulfur subunit of mitochondrial respiratory complex II, which also participates in oxygen sensing and response^12^,^13^,^14^. Mutations in *SDH* genes are associated with both hereditary and non-hereditary paraganglioma and pheochromocytoma, renal carcinoma and gastrointestinal stromal tumours^15^. More recently, we found that hypoxia (1% O_2_) enhances the C>U editing of *SDHB* RNA at c.136 in monocytes, with an editing level of ∼18% observed for monocyte-enriched PBMCs (MEPs) after 48 h of hypoxia^16^. Monocytes infiltrate tumours, atheromatous plaques and sites of infection and inflammation, which are characterized by micro-environmental hypoxia. C>U RNA editing of *SDHB* may therefore represent a hypoxia-adaptive mechanism that may have implications for the pathogenesis of chronic inflammatory diseases.

To identify additional C>U RNA editing events in monocytes and monocyte-derived macrophages (MEPs), we analyse their whole transcriptome RNA sequences. We show that transcripts of hundreds of genes including those implicated in viral pathogenesis and Alzheimer's disease are targets of editing in monocytes and macrophages. Such editing is regulated by oxygen, interferons (IFNs) and also during macrophage polarization. Most importantly, we demonstrate that APOBEC3A, which belongs to the APOBEC3 family of CDAs, is an RNA-editing enzyme. These findings significantly expand our understanding of C>U RNA editing and open new avenues of inquiry on the role of *APOBEC3* genes in viral and chronic diseases.

## Results

### *SDHB* RNA editing in IFN-treated MEPs and M1 macrophages

**Figure 1:**
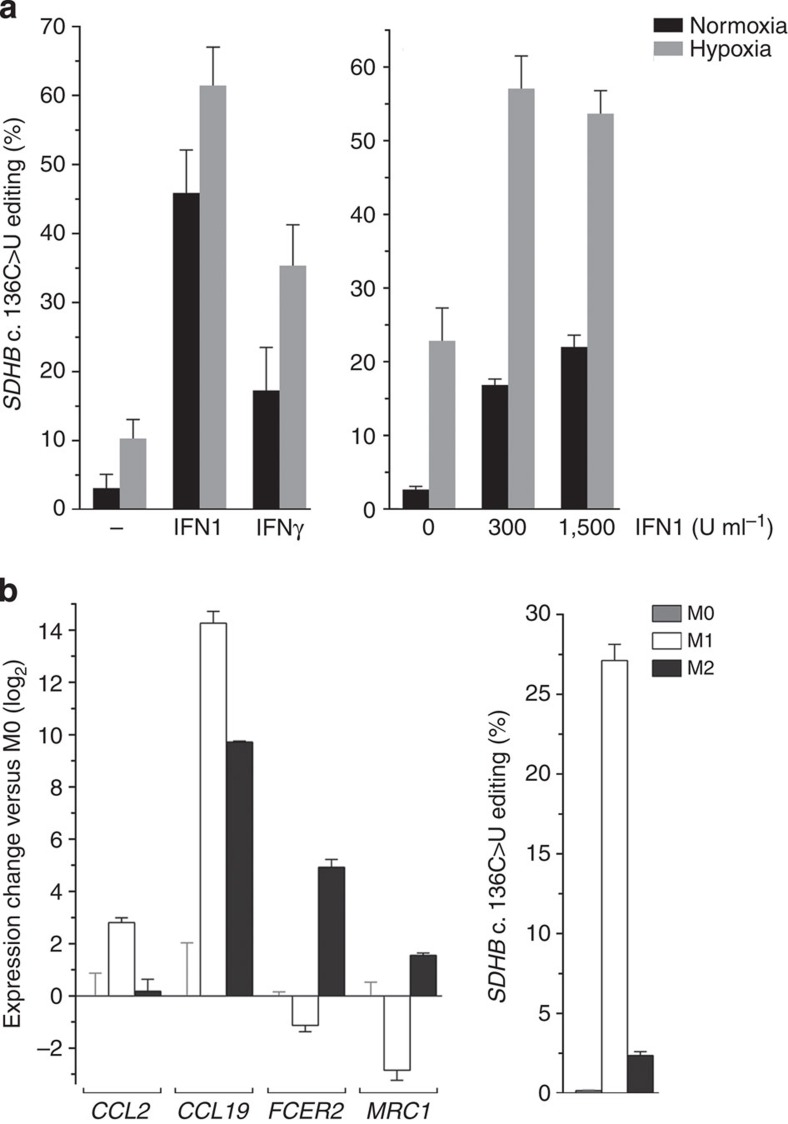
*SDHB* c.136C>U RNA editing in IFN-treated MEPs and M1 macrophages (**a**) Mean and its s.e. (*n*=3) are shown on left for editing levels in MEPs optionally treated with IFN1 (600 U ml^−1^), IFNγ (200 U ml^−1^) and hypoxia (1% O_2_) for 24 h. The additive induction of *SDHB* c.136C>U RNA editing by the IFNs and hypoxia is also depicted on right. Matched MEPs of seven individuals were cultured under normoxia or hypoxia with 0, 300 or 1,500 U ml^−1^ IFN1 for 24 h. Mean and its s.e. (*n*=7) for editing levels in the cells are shown. Editing level in cells treated with both hypoxia and IFN1 was higher than in cells treated with only hypoxia or IFN1 (Wilcoxon test *P*<0.02, for both concentrations of IFN1). (**b**) M1 and M2 macrophages were generated from unpolarized M0 macrophages derived from CD14+ monocytes isolated from peripheral blood of three individuals. Mean and range (*n*=3) of expression of genes for markers of M1 and M2 polarization and *SDHB* c.136C>U editing levels in the cells are depicted. Gene expression was quantified by RT–PCR and normalized to that of *ACTB*.

Similar to hypoxia, an IFN-rich microenvironment is another factor that monocytes are exposed to during inflammation. IFNs also upregulate expression of APOBEC3 CDAs^17^, candidate enzymes that may be responsible for the *SDHB* c.136C>U RNA editing observed in monocytes. We therefore examined whether IFNs induce *SDHB* c.136C>U RNA editing. As shown in the left panel of Fig. 1a, treatment of MEPs with type 1 IFN (IFN1; 600 U ml^−1^) or IFNγ (200 U ml^−1^) for 24 h induced *SDHB* c.136C>U RNA editing in MEPs, both in normoxia and hypoxia under 1% O_2_ (Mann–Whitney *U*-test *P*<0.01, comparing untreated and IFN-treated samples). The editing level in normoxic or hypoxic MEPs was increased ∼6-fold by IFN1 and ∼3-fold by IFNγ, suggesting that the induction of RNA editing with IFN1 was higher than with IFNγ (Wilcoxon rank sum test *P*<0.03, comparing samples regardless of hypoxia treatment). An additive effect of IFNs and hypoxia on *SDHB* c.136C>U RNA editing was observed and this was confirmed in an independent experiment in which matched MEPs of seven individuals were cultured under normoxia or hypoxia with 0, 300 or 1,500 U ml^−1^ IFN1 for 24 h. Editing level in cells treated with both hypoxia and IFN1 was higher than in cells treated with only hypoxia or IFN1 (Fig. 1a, right panel; Wilcoxon test *P*<0.02, for both concentrations of IFN1).

IFNγ is an inducer of M1 (pro-inflammatory) polarization of macrophages, which are derived from monocyte precursors. We therefore examined and compared *SDHB* c.136C>U RNA editing in basal unpolarized (M0), M1 and M2 macrophages. M0 cells were derived *in vitro* from CD14+ peripheral blood monocytes and matched M1 and M2 macrophages were generated from the M0 cells by treatment with IFNγ and lipopolysaccharides (LPS) and interleukin-4, respectively. The *SDHB* RNA editing was found to be absent in M0 macrophages but occurred at an average level of ∼27% in M1 cells (Fig. 1b, right panel). The editing level was significantly lower in M2 macrophages (∼2%), suggesting a strong induction of editing in macrophages by M1 but not M2 polarization.

### Widespread RNA editing in hypoxic MEPs and M1 macrophages

To investigate whether hypoxia affects editing of RNAs other than *SDHB* in MEPs, we performed RNA sequencing of matched normoxic and hypoxic MEPs of three healthy individuals. We also examined whole transcriptome RNA sequencing data obtained by Beyer *et al*.^18^ for matched M1 and M2 macrophages generated *in vitro* from peripheral blood monocytes of three individuals, to determine whether M1 macrophage polarization differentially affects editing of other RNAs besides *SDHB*. Such comparison of whole transcriptomes of paired samples to identify RNA editing is less likely to falsely identify sequencing and mapping artefacts or genome sequence variations as RNA-editing events.

About 84%–90% and 94%–97% of RNA sequencing reads of the MEPs and macrophages, respectively, could be uniquely mapped to the UCSC hg19 reference human genome (Supplementary Tables 1–3). Calls made by the mapped reads for the reference base or a variation were counted along the genome and paired count data were evaluated with the inverted β-binomial test^19^ to identify genome positions at which the base variation level was differentially affected by hypoxia or M1 polarization with >2-fold change in either direction, with a *q*-value of <0.05 and a higher intra-group mean variation level of ⩾5%. The type of RNA editing at a genome position was surmised from the base variation and the gene-coding chromosome strand at the position. The candidate RNA-editing sites were filtered to remove probable false positives. Filtering criteria included identification of the site with a separate read-mapping software and location of the site within a known RefSeq gene (Methods and Supplementary Table 4).

**Figure 2:**
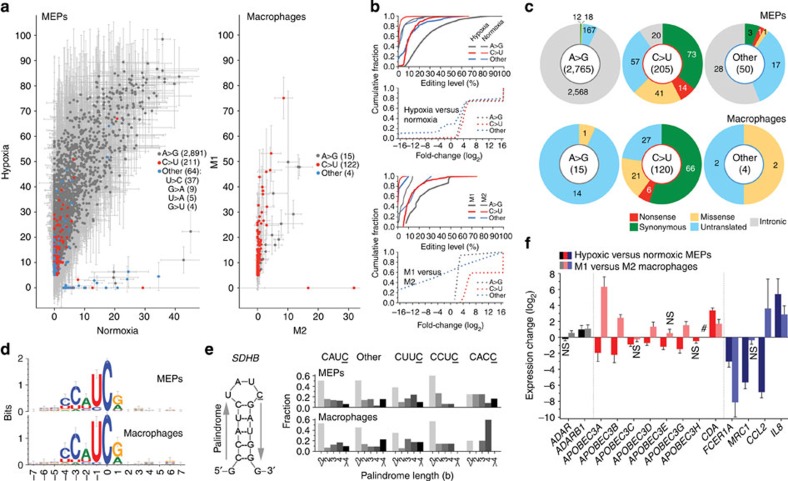
RNA editing in MEPs and macrophages (**a**) Mean and range of RNA-editing levels (%) at sites identified by comparing transcriptome sequences of three pairs of hypoxic and normoxic MEPs, or M1 and M2 macrophages for differential RNA editing under hypoxia or M1 polarization. (**b**) Cumulative frequency plots of mean editing levels and fold-change effects of hypoxia or M1 polarization on editing level, by type of RNA editing. Fold-change values were estimated with the inverted β-binomial test and their absolute values are capped at 10^4^. (**c**) Distributions for editing sites in coding RNAs of gene feature and effect of editing on amino acid coding, by type of RNA editing. (**d**) Logos indicating sequence conservation and nucleotide frequency for sequences bearing C>U editing sites (at position 0) with a higher editing level in hypoxic compared with normoxic MEPs (*n*=206) or M1 compared with M2 macrophages (*n*=122); mean and 95% confidence interval (CI) of relative entropy values are also plotted. (**e**) Stem-loop structure in *SDHB* RNA with the c.136C>U editing site underlined and 5-b palindromes forming the stem indicated. Histograms depict the distributions of flanking palindrome length by sequence at −3 to 0 positions for the sites whose sequence logos are shown in **d**. (**f**) Effect of hypoxia or M1 polarization on transcript levels of genes that are expressed in MEPs or macrophages and code for ADAR and cytidine deaminase enzymes and some markers of M1 (*FCER1A* and *MRC1*) or M2 (*CCL2* and *IL8*) macrophage polarization. Mean and range (*n*=3) are shown; NS, not significant (FDR ⩾0.05, edgeR likelihood ratio test); *#*, not expressed; genes not marked NS or *#* are differentially expressed with FDR <0.05.

Putative RNA editing was found to be up- and downregulated respectively at 3,137 and 29 sites by hypoxia in MEPs, and respectively at 139 and 2 sites by M1 compared with M2 macrophage polarization (Fig. 2a,b and Supplementary Data 1). Editing in MEPs was of A>I (A>G) and C>U types at 91.3% and 6.6% of the sites, respectively, whereas these two types of editing respectively occurred at 10.6% and 86.5% of the sites in macrophages. A>G editing occurred at an overwhelming majority of the sites in MEPs, but only 1.0% of the A>G sites were in coding exons, causing 18 non-synonymous and 12 synonymous codon changes (Fig. 2c and Supplementary Table 5). This is consistent with the known targeting of A>G RNA editing to non-coding sequences^4^. On the other hand, 61.7% of the total 211 C>U sites (in 199 genes) were in coding exons, causing 55 non-synonymous and 73 synonymous codon changes. C>U editing accounted for 73.3% of all non-synonymous editing upregulated by hypoxia in MEPs. In macrophages, 77.9% of the total 122 C>U sites (in 116 genes) were in coding exons, causing 27 non-synonymous and 66 synonymous codon changes.

The average editing levels in hypoxic MEPs were >10% and >20%, respectively, for 93 (45%) and 25 (12%) of the 206 C>U sites for which editing was upregulated by hypoxia. In normoxic MEPs, the levels were <1% and <5%, respectively, for 162 (79%) and 202 (98%) of the 206 sites (Fig. 2b). Average C>U editing level in M1 macrophages was >10% and >20%, respectively, for 62 (51%) and 24 (20%) of the 122 sites. In contrast, levels in M2 cells were <1% and <5%, respectively, for 105 (86%) and 121 (99%) sites (Fig. 2b). Notably, 55 C>U RNA editing sites were shared by and upregulated in both the hypoxic MEPs and M1 macrophages. Editing of none of the 122 sites in M1 macrophages was downregulated by hypoxia in MEPs. Ontology analysis of C>U RNA-edited genes of both MEPs and macrophages revealed enrichment for genes encoding for catalytic activities and for genes in integrin-mediated signalling, and Alzheimer's, Huntington's and Parkinson's disease pathways (Supplementary Table 6).

### Sequence and structural contexts of C>U RNA-editing sites

C>U editing sites were most commonly present within a CCAUCG sequence motif (edited site underlined), with CAUC and its CACC, CCUC, CUUC and UAUC 1-nucleotide (nt) variants present for ∼79% and 85% of the editing sites of MEPs and macrophages, respectively (Fig. 2d and Supplementary Data 1). As the UAUC motif containing the *SDHB* c.136 nucleotide was flanked by palindromic sequences (Fig. 2e), we examined other C>U RNA-editing sites, to determine whether the edited Cs in these were also flanked by palindromic sequences. Approximately 51% and 52% of all edited NNNC sequences of MEPs and macrophages, respectively, were found to be flanked by short palindromic sequences of 2–7 nt (median=2 and 3 nt, respectively; Fig. 2e and Supplementary Data 1). Examination of minimum free-energy structures^20^ of 60 nt sequences bearing the edited C in the middle showed that the C residue was present in the loop of a stem-loop structure for 72% and 67% of the sites of MEPs and macrophages, respectively (Supplementary Data 1). These observations suggest that C>U RNA editing in MEPs and macrophages is catalysed by CDA(s) with particular target sequence and structure preference.

### Validation of site-specific C>U RNA editing in MEPs

**Figure 3:**
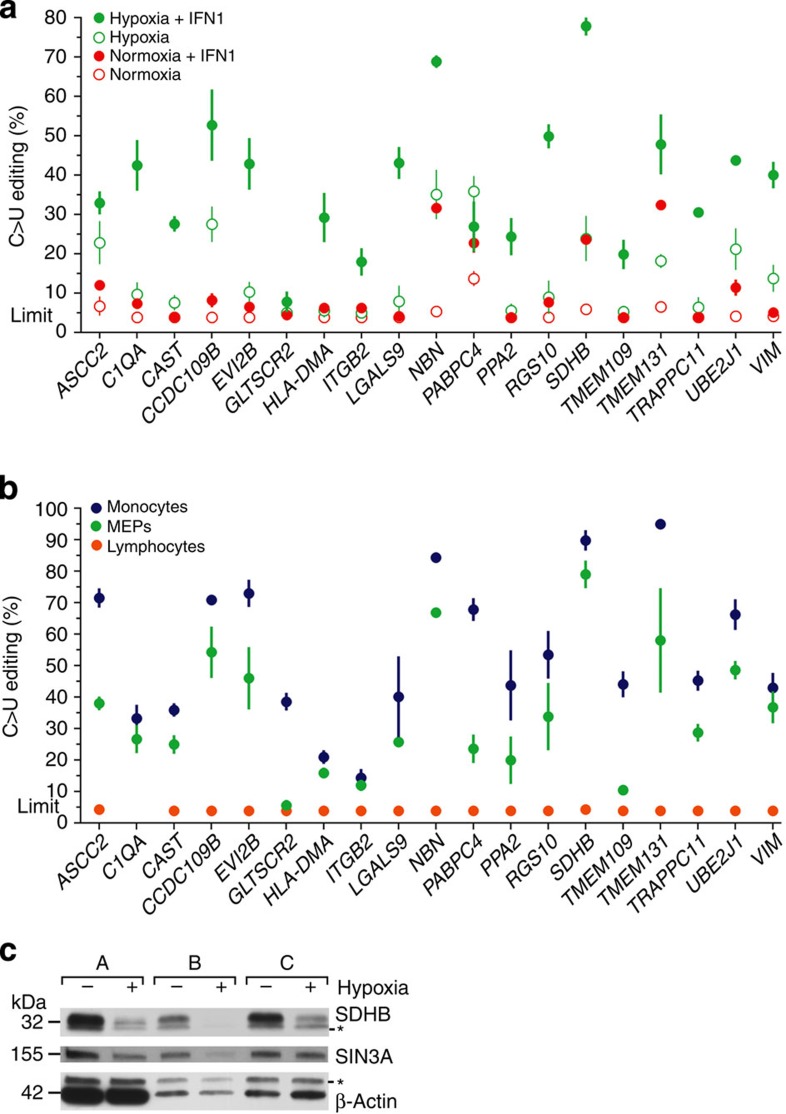
C>U RNA editing induced in MEPs and monocytes by hypoxia and IFN1. (**a**) Site-specific C>U RNA editing for 19 genes of MEPs of three individuals was quantified by Sanger sequencing of RT–PCR products. MEPs were optionally treated with hypoxia and/or 600 U ml^−1^ IFN1 for 24 h. (**b**) Editing of the sites was also similarly examined in hypoxia- and IFN1-treated MEPs of another three individuals and in lymphocytes and CD14+ monocytes isolated from the MEPs. Because of absent or low gene expression, a *C1QA* RT–PCR product could not be obtained for any of the three lymphocyte isolates. Sanger sequence chromatograms for the three monocyte and two of the lymphocyte isolates are shown in Supplementary Fig. 3c. Site-specific C>U RNA editing in the monocytes and lymphocytes for 12 other genes is depicted in Supplementary Fig. 3b. Mean and its s.e. (*n*=3) are shown in both panels. The detection limit for editing (5% level) is indicated. Samples without detectable editing were assigned a value of 3.8%. (**c**) SDHB and SIN3A protein levels in whole-cell lysates (20 μg protein) of monocytes isolated from normoxic and hypoxic MEPs of a separate set of three donors. Non-specific signals of the western blots are indicated by asterisk (*).

Thirty-three non-synonymous C>U RNA-editing sites (in 33 genes) that were identified in the analysis of RNA sequencing data (Fig. 2a) were chosen for experimental validation of site-specific editing by Sanger sequencing of reverse transcriptase–PCR (RT–PCR) products. Eighteen of the 33 sites were identified in MEPs, 3 in macrophages and 12 in both (Table 1). RNA editing for 31 of the 33 genes, including the three exclusively identified in macrophages, could be experimentally validated in MEPs (Table 1). The RNA-editing level for 19 genes was quantified in MEPs of three donors. Editing for none of the genes was observed in normoxic MEPs, but was seen for all in MEPs treated with hypoxia with or without IFN1 (Fig. 3a and Supplementary Fig. 1). The additive effect of hypoxia and IFN1 on C>U RNA editing previously observed for *SDHB* (Fig. 1a) was also noticeable in the Sanger-sequencing analyses for site-specific RNA editing of 18 other genes (Fig. 3a); the editing levels observed with combined hypoxia and IFN1 treatment (mean=38.2%) were significantly higher (Wilcoxon paired ranks test *P*<0.005) than the sum of those with IFN1 (mean=10.8%) or hypoxia (mean=14.5%) alone. Editing levels did not significantly differ between hypoxia and IFN1 treatments (analysis of variance test *P*>0.05). Sanger sequencing of PCR-amplified genomic DNA fragments of hypoxia- and IFN1-treated MEPs did not reveal C>T nucleotide variation at the editing site for any of the 23 genes that were examined (Supplementary Fig. 2).

MEPs contain both monocytes and lymphocytes. To determine the RNA-editing levels of the 31 experimentally validated genes in these individual cell types, Sanger sequencing of RT–PCR products of monocyte and lymphocyte isolates (Supplementary Fig. 3a) of hypoxia- and IFN1-treated MEPs of another three individuals was performed (Fig. 3b and Supplementary Fig. 3b,c). Editing levels in monocytes were more than in their parent MEPs, and >20% for 29 genes and >80% for 5 (*TMEM131*, 95%; *SDHB*, 90%; *PCGF3*, 90%; *NBN*, 84%; and *RNH1*, 83%). In lymphocytes, RNA editing was seen for only two of the 34 genes (*FAM89B* and *RHN1*, ∼8% level for each), suggesting that most of the differential C>U RNA editing in MEPs occurred in the monocytes.

For two of the transcripts for which the editing results in a nonsense codon change, *SDHB* (NCBI reference sequence NM_003000, exon 2:p.R46X) and *SIN3A* (NM_001145357, exon 20:p.Q1197X), the effect of hypoxia-induced C>U RNA editing on protein level was examined by immunoblotting assays of whole-cell lysates of monocytes isolated from normoxic or hypoxic MEPs of three donors in a separate experiment. As shown in Fig. 3c, hypoxia treatment of MEPs resulted in a significant reduction of both SDHB (280 amino acid residues, NCBI reference sequence NP_002991) and SIN3A (1,273 residues, NP_001138829) in monocytes. Hypoxia also reduced *SDHB* and *SIN3A* RNA levels by an average of 4.7- and 1.6-fold in these three CD14+ monocyte samples, as tested by quantitative RT–PCR (normalized against the *B2M* gene). Although this reduction could be at the transcriptional level, it could also be a result of posttranscriptional processes such as nonsense-mediated decay and microRNA targeting of the transcripts because of the sequence change resulting from their editing.

### *APOBEC3A* expression is associated with *SDHB* RNA editing

Next, we examined whether expression of any CDA gene(s) was associated with *SDHB* c.136C>U RNA editing in monocytes and macrophages*. CDA* and the seven *APOBEC3* genes were identified as expressed in RNA sequencing data of MEPs and only *CDA* expression was upregulated by hypoxia (Fig. 2f). Expression of *APOBEC3A*, the only *APOBEC3* gene that is expressed at a higher level in monocytes compared with lymphocytes^21^, was downregulated by hypoxia. Expressions of *CDA* and four *APOBEC3* genes (*A*, *B*, *D* and *G*) were upregulated in M1 compared with M2 macrophages, with *APOBEC3A* upregulation being the highest (∼67-fold), whereas *AID* and *APOBEC1*, *2* and *3H* were not expressed in macrophages (Fig. 2f). Upregulation of *APOBEC3A* expression by IFN1, as has been shown by others^17^, was seen in normoxic as well as hypoxic MEPs; IFN1 did not upregulate *CDA* expression and upregulated *APOBEC3G* expression only under normoxia (Supplementary Fig. 4). Examination of changes in expression of CDA genes in MEPs by hypoxia and IFN1, and in macrophages by M1 compared with M2 polarization, therefore suggested APOBEC3A and CDA as possible mediators of inducible C>U editing in MEPs and macrophages.

To further understand the association of CDA gene expression with *SDHB* c.136C>U RNA editing, we evaluated RNA sequencing data in the Cancer Genome Atlas (TCGA) for three randomly chosen cancers, primary head and neck squamous cell carcinoma, lung adenocarcinoma and secondary skin cutaneous melanoma. As tumours contain immune cells and can have hypoxic regions, we hypothesized that some degree of *SDHB* c.136C>U variation may be noticeable in the RNA sequences of the TCGA samples. Somatic *SDHB* c.136C>T mutation has not been identified in any TCGA sample for these cancers (data release 17 of the International Cancer Genome Consortium^22^).

**Figure 4:**
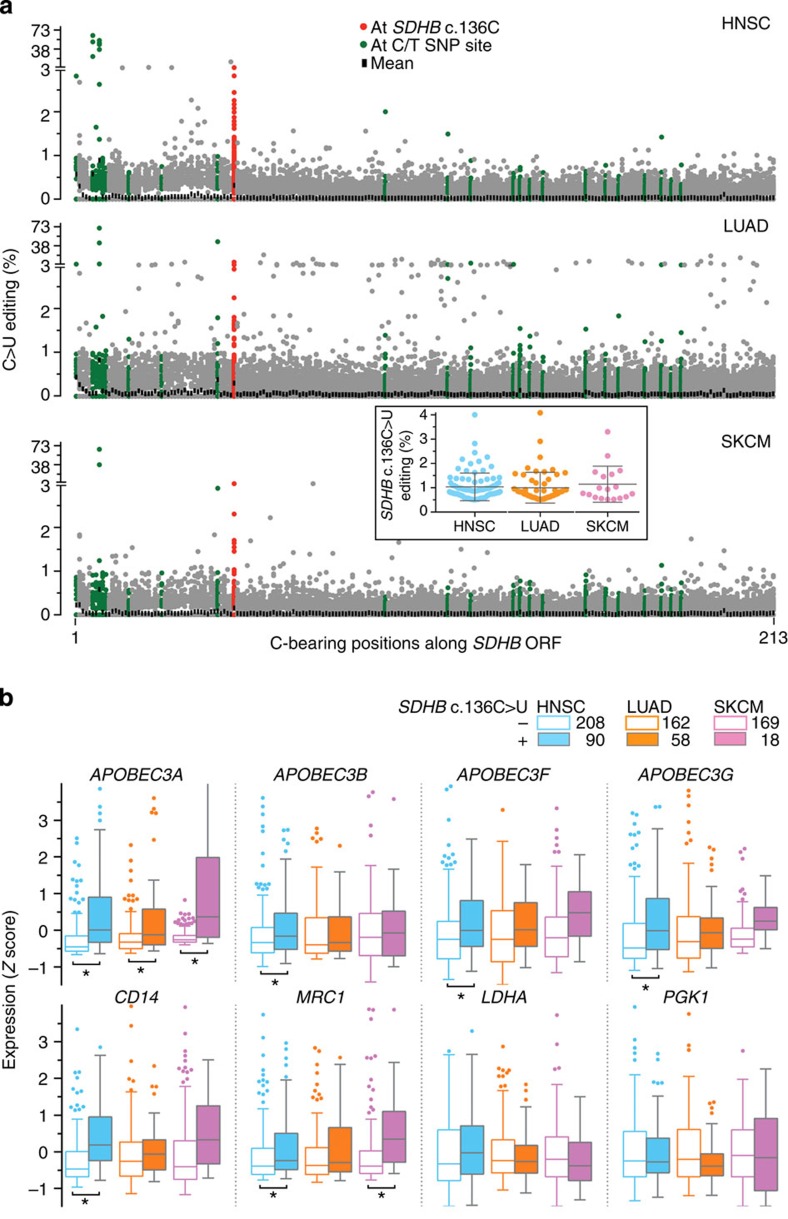
Association of *APOBEC3A* gene expression with *SDHB* c.136C>U RNA editing in tumour samples of TCGA (**a**) C>U RNA editing was estimated from RNA-sequencing data for primary head and neck squamous cell carcinoma (HNSC, *n*=298), lung adenocarcinoma (LUAD, *n*=220) and secondary skin cutaneous melanoma (SKCM, *n*=187) tumours. Editing levels at all 213 C-bearing positions along *SDHB* ORF are plotted for every tumour. Mean levels at the positions (black), the c.136C site (red) and known C/T single-nucleotide polymorphism sites (green) are indicated. Inset shows *SDHB* c.136C>U editing levels and their mean and s.d. for tumours identified as positive for the editing. (**b**) Tukey's plots of expression of some *APOBEC3* (*A3*) and hypoxia- (*LDHA* and *PGK1*) and macrophage-associated (*CD14* and *MRC1*) genes among *SDHB* c.136C>U editing-positive and -negative tumours. Error bars denote 25th percentile −1.5 × interquartile range (IQR) and 75th percentile+1.5 × IQR values. Group sizes are noted in the legend. *FDR <0.05 (edgeR exact test for differential expression).

The scrutiny of TCGA's RNA sequencing data for the tumour tissues indicated putative C>U RNA editing of *SDHB* open reading frame (ORF) at c.136, but at no other site, in 30.2%, 26.4% and 9.6% of 298 primary head and neck squamous cell carcinoma, 220 lung adenocarcinoma and 187 secondary skin cutaneous melanoma cases that were examined, respectively (Fig. 4a). The editing levels were low (∼1%), suggesting that it occurred only in a fraction of the cells of the tumours. Whole-exome sequencing data for all eight tumours with editing level >2.25% showed complete absence of any sequence variation at c.136 at the genomic level (depth of coverage for *SDHB* c.136 ranging from 40 to 111, with mean=77). Comparison of gene expression between the editing-positive and -negative samples showed that *APOBEC3A* was the only CDA gene whose expression was upregulated in the editing-positive samples in all three cancers. Consistent differential expression of common hypoxia- or monocyte/macrophage-associated genes across all three cancers between editing-negative and -positive samples was not seen (Fig. 4b and Supplementary Tables 7 and 8).

### *APOBEC3A* overexpression causes C>U RNA editing in 293T cells

**Figure 5:**
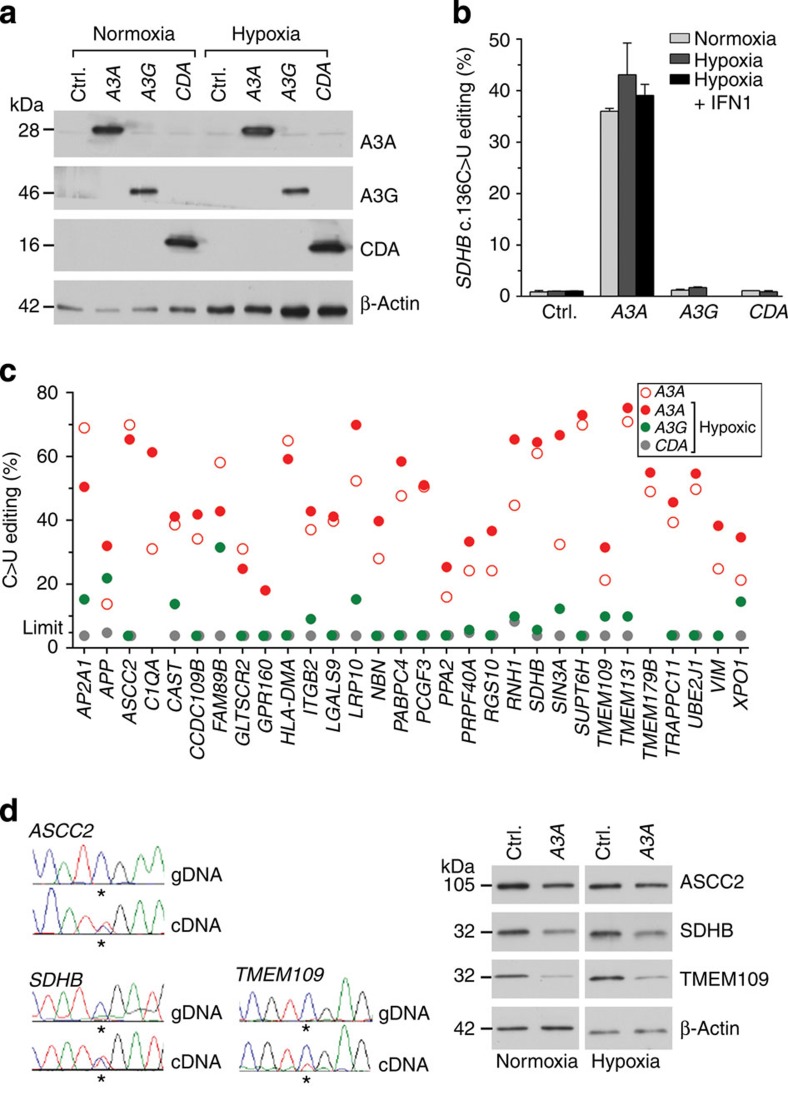
APOBEC3A induces C>U RNA editing in 293T transfectants. (**a**) Immunoblots showing APOBEC3A (*A3A*), APOBEC3G (*A3G*) and CDA proteins in whole-cell lysates (20 μg protein) of 293T cells transiently transfected with an empty vector (*Ctrl.*, control) or DNA constructs for expression of A3A, A3G or CDA proteins. (**b**) *SDHB* c.136C>U RNA editing in the 293T transfectants, which were optionally treated with hypoxia and/or 600 U ml^−1^ type I IFN (*IFN1*). Mean and range for *n*=3 are shown. (**c**) Estimation of site-specific C>U RNA editing by Sanger sequencing of RT–PCR products for 30 genes in the transfectants (*n*=1). The detection limit for editing (5% level) is indicated. Samples without detectable editing were assigned a value of 3.8%. Chromatograms for 19 genes are shown in Supplementary Fig. 5. Chromatograms of good quality could not be obtained for *C1QA* and *TMEM179B* for the *A3G* and *CDA* transfectants, and for the *GPR160* site for the normoxic *A3A* transfectant. (**d**) Chromatograms of genomic DNA (*gDNA*) and cDNA PCR products of normoxic *A3A* transfectants, indicating C>U RNA editing without C>T genomic change at positions marked with * for *ASCC2*, *SDHB* and *TMEM109*. Immunoblots showing ASCC2, SDHB and TMEM109 proteins in whole-cell lysates (20 μg protein) of control or *A3A* transfectants on the right indicate reduced protein expression in association with A3A-induced stop codons in RNA. Only a single band of signal, which corresponded to a protein of full length, was seen in all three immunoblots.

As noted above, the expression of *APOBEC3A* or *CDA* positively correlated the most with C>U RNA editing in cancer tissues, MEPs or macrophages (Supplementary Table 8). To test whether *SDHB* c.136C>U RNA editing can be induced by these two proteins, or by *APOBEC3G* whose expression is upregulated by M1 macrophage polarization, their complementary DNAs were exogenously expressed in the human 293T embryonic kidney cell line in which all three proteins were undetectable (Fig. 5a). Transient transfection of 293T cells for exogenous expression of *APOBEC3A*, but not *APOBEC3G* or *CDA*, induced *SDHB* c.136C>U RNA editing in the cells (Fig. 5b). Treatment of transfectants for 24 h with hypoxia (1% O_2_) but not IFN1 (600 U ml^−1^) mildly enhanced this editing (Fig. 5b). Previous studies have shown that intronic sequences are essential for A>I RNA editing, but not for APOBEC1-mediated C>U editing of *APOB*, which occurs in the nucleus after the *APOB* pre-mRNA has been spliced^9^,^23^. We found evidence for c.136C>U RNA editing of transcripts generated *in vivo* from a co-transfected, intron-less *SDHB* ORF cDNA expression construct in *APOBEC3A* transfectants, indicating that intronic sequences are not required for APOBEC3A-mediated RNA editing (Supplementary Fig. 6).

Sanger sequencing of RT–PCR products of the 293T transfectants showed that exogenous APOBEC3A, but not CDA, also caused site-specific C>U RNA editing for 30 genes for which RNA editing was previously validated for MEPs (editing for *EVI2B* could not be examined because of low gene expression; Fig. 5c and Supplementary Fig. 5). This suggests that APOBEC3A mediates the transcriptome-wide C>U RNA editing that was noted for MEPs and macrophages (Fig. 2a). For most of the gene transcripts, hypoxia mildly increased the RNA-editing levels from an average level of 42% to 49% (Wilcoxon paired ranks test *P*=0.002; *n*=29). Sanger sequencing of PCR-amplified genomic DNA fragments of transfectants did not reveal C>T nucleotide variation at the editing site for any of the 23 genes that were examined (Supplementary Fig. 2 and Fig. 5d). We tested the effect of APOBEC3A-mediated RNA editing on the protein expression of three genes. Western blot assays of whole-cell lysates of the transfectants for three proteins, ASCC2, SDHB and TMEM109, whose RNA transcripts were predicted to have p.R121X (in exon 4; NCBI reference sequence NM_032204, which encodes a protein of 757 aa), p.R46X (in exon 2; NM_003000, 280 aa) and p.R37X (in exon 2; NM_024092, 243aa) nonsense codon changes, respectively, because of RNA editing showed that exogenous APOBEC3A expression reduced levels of the proteins in 293T cells (Fig. 5d). In a separate RNA-sequencing experiment, exogenous APOBEC3A expression in 293T cells was found not to affect *SDHB* RNA level in comparison with control transfectants, whereas it mildly but significantly affected *ASCC2* and *TMEM109* transcript levels, with fold-change values of ∼1.1 and 0.8, respectively. These results suggest that stop codons introduced by RNA editing may reduce wild-type protein levels.

**Figure 6:**
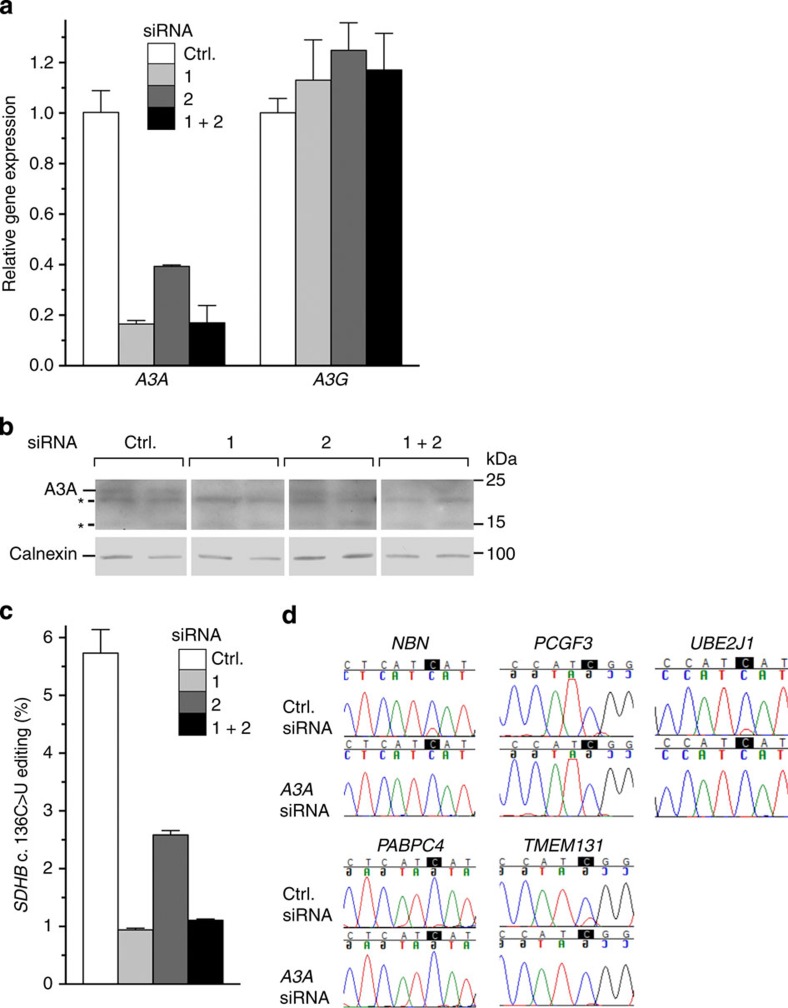
Knockdown of *APOBEC3A* (*A3A*) reduces C>U RNA editing in M1 macrophages (**a**) *A3A* and *APOBEC3G* (*A3G*) gene expression in M1 macrophages that were transfected with a nonspecific (*Ctrl.*) or either one (*1*, *2*) or equimolar mix (*1+2*) of two *A3A*-specific siRNAs at 100 nM concentration. Gene expression measurements are normalized to that for *ACTB*. (**b**) Immunoblot for A3A protein (23 kDa) of whole-cell lysates (10 μg protein) of two of each set of three replicate transfectants. Nonspecific signals are indicated by an asterisk (*). The signal for calnexin, a house-keeping protein, indicates total protein. (**c**) *SDHB* c.136C>U RNA-editing levels in the siRNA transfectants, which are determined by RT–qPCR. (**d**) Sanger sequence chromatogram traces of amplified cDNA fragments, indicating reduced site-specific RNA editing for five other genes in *A3A*-specific siRNA *1* compared with *Ctrl.* transfectants. Mean and range (*n*=3) are shown for **a** and **c**.

Notably, exogenous APOBEC3G also caused low-level, site-specific RNA editing for 11 genes in 293T transfectants (Fig. 6a); editing levels were highest for *FAM89B* and *APP*, for both of which the edited cytidine residue occurs in a CC sequence context that is known to be preferred by APOBEC3G for DNA deamination^24^.

### APOBEC3A knockdown reduces RNA editing in M1 macrophages

To validate that APOBEC3A mediates *SDHB* c.136C>U RNA editing in M1 macrophages (Fig. 1b), we transfected M0 macrophages with small interfering RNA (siRNA) at 100 nM to knock down *APOBEC3A* RNA, induced their M1 polarization after a day and examined the M1-polarized cells after another 24 h. Transfection of cells with either of the two different siRNAs predicted to target *APOBEC3A*, or their equimolar mix, led to a significant reduction in *APOBEC3A* transcript and APOBEC3A protein levels compared with cells transfected with a control siRNA that is not predicted to target *APOBEC3A* (Fig. 6a,b). There was no effect on *APOBEC3G* RNA level in the cells, suggesting that the knockdown was gene specific (Fig. 6a,b). Reduction of *APOBEC3A* RNA level was associated with a significant reduction of *SDHB* c.136C>U RNA editing (Fig. 6c), indicating that APOBEC3A is a major determinant of this editing in M1 macrophages. Sanger sequencing of RT–PCR products was used to evaluate site-specific C>U editing level for transcripts of five other genes for which RNA editing in M1 macrophages had been noted in the analysis of transcriptome sequencing data (Fig. 2a). Examination of the sequence chromatograms showed that macrophages transfected with an siRNA predicted to target *APOBEC3A* had a lower level of RNA editing for all five genes compared with cells that were transfected with the control siRNA (Fig. 6d).

### RNA editing by APOBEC3A variants and retrotransposition

**Figure 7:**
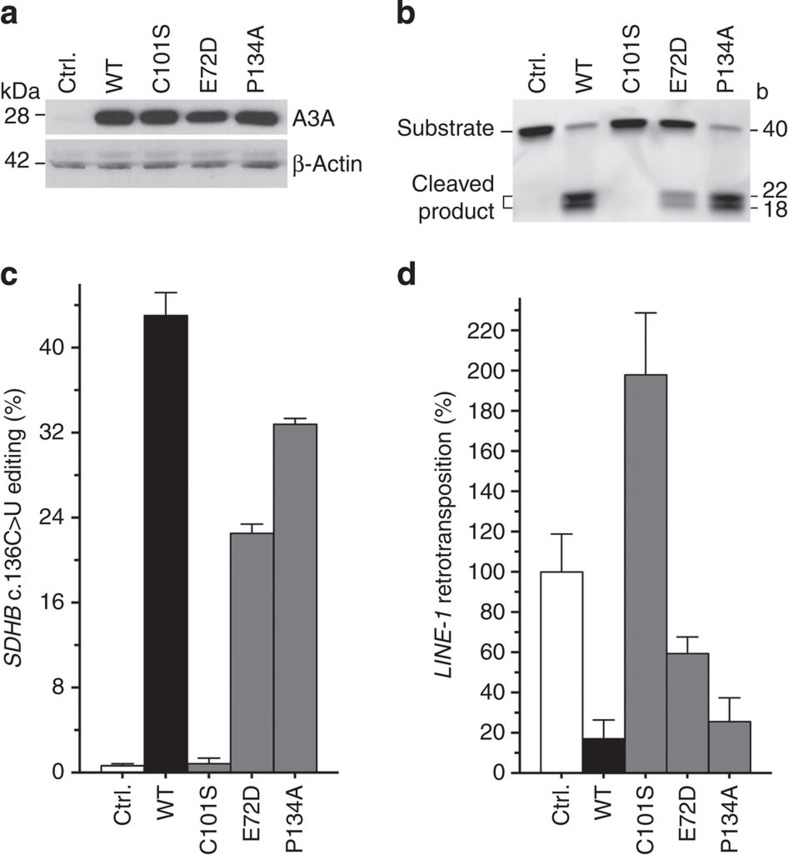
Activity of APOBEC3A (A3A) mutants in 293T transfectants (**a**) A3A protein level in whole-cell lysates (20 μg protein) of cells transfected with an empty vector (*Ctrl.*) or expression constructs for wild-type (*WT*) A3A or its C101S, E72D or P134A variants. (**b**) Cytidine deamination activity of the transfectant lysates was examined in an *in vitro* reaction with a 5′ fluorescent dye-labelled ssDNA substrate of 40 bases (b). C>U deamination of the single cytidine residue of the substrate at position 23 followed by deglycosylation of the uridine and subsequent cleavage of the product at the abasic site was evaluated by electrophoresis of reactions of one hour duration on a polyacrylamide gel, whose fluorographic image is shown. (**c**) *SDHB* c.136C>U RNA editing in the transfectants. (**d**) Retrotransposition of a human *LINE-1* element in a separate set of 293T transfectants. Retrotransposition, relative to the *Ctrl.* transfectant, was assessed with a luciferase reporter-based assay and is quantified as the ratio of firefly and Renilla luciferase activities. Mean and range (*n*=3) are shown for **c** and **d**.

The C101 residue of APOBEC3A is critical for binding of zinc and the C101S APOBEC3A mutant completely lacks deamination activity against cytidines of ssDNA *in vitro*^25^,^26^. As expected, cell lysates of the 293T transfectants exogenously expressing this mutant (Fig. 7a) did not cause deamination of the single cytidine residue of an ssDNA 40-mer (Fig. 7b) in a previously established assay.^27^ To test whether C101 residue is essential for the observed RNA editing, we transfected 293T cells with the mutant cDNA. *SDHB* c.136C>U RNA editing or site-specific C>U RNA editing for five other examined genes for which editing was observed in transfectants expressing the wild-type APOBEC3A was abolished in the C101S APOBEC3A transfectant (Fig. 7c and Supplementary Fig. 5). The E72D and P134A variants of APOBEC3A were previously shown to variably impair the ssDNA deamination activity of the wild-type enzyme^26^. We found that whole-cell lysate of 293T transfectant of E72D, but not P134A, was moderately impaired in the ssDNA deamination assay (Fig. 7a,b). Unlike for C101S, the E72D variant was capable of C>U RNA editing of transcripts for *SDHB* and five other genes that were examined, although to lesser levels than the wild-type protein (Fig. 7c and Supplementary Fig. 5). The *SDHB* RNA editing level in transfectants of the P134A variant was ∼80% of that of transfectants expressing the wild-type APOBEC3A (Fig. 7c). These results suggest that the catalytic activity required for DNA deamination by APOBEC3A is also important for RNA editing.

APOBEC3A suppresses retrotransposition in cell-based assays and this suppression is dependent on its ssDNA cytidine deaminating catalytic integrity (see discussion). To test whether RNA editing and retrotransposition-suppressing functions of APOBEC3A are linked, we tested the effect of mutations on *LINE1* retrotransposition, using a previously described cell-based assay^26^. We found that the ability of the E72D, C101S and P134A variants to inhibit retrotransposition paralleled their RNA-editing activities (Fig. 7d). These findings indicate that mutations in E72, C101 and P134 residues of APOBEC3A affect the protein's ssDNA and RNA deamination, and anti-*LINE1* retrotransposition activities in a similar manner.

### *In vitro* deamination of *SDHB* RNA and ssDNA by APOBEC3A

**Figure 8:**
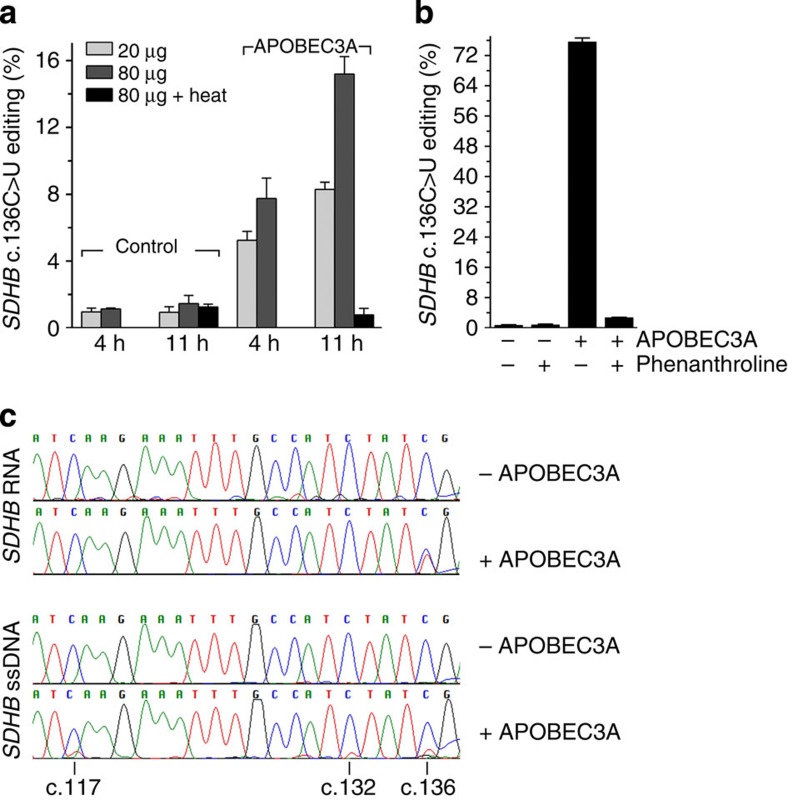
*In vitro* cytidine deamination of *SDHB* RNA and ssDNA by APOBEC3A (**a**) c.136C>U editing of an ∼1.1 kb exogenous *SDHB* ORF RNA by whole-cell lysates of control or *APOBEC3A* 293T transfectants. Duration of the deamination reactions and amount of lysate protein in them are noted. For some reactions, lysates were pre-heated at 85 °C for 15 min. (**b**) c.136C>U editing of the RNA by 10 μM purified *C*-His_6_-tagged APOBEC3A protein. The reactions had 180 amole *SDHB* RNA and 100 nM ZnCl_2_. (**c**) Sanger sequence chromatogram traces of PCR amplified products of *SDHB* deamination reactions that had either 180 amole of ∼1.1 kb *SDHB* RNA or 100 amole of *SDHB* ssDNA of 120 b as substrate. APOBEC3A protein was present in the+reactions at 5 and 20 μM in the reaction with RNA and DNA substrate, respectively. Reactions for **b** and **c** conducted for two hours at 37 °C. Mean and range (*n*=3) are shown in **a** and **b**, respectively.

The various observations thus far noted suggest that APOBEC3A can deaminate cytidines in RNA. To demonstrate that APOBEC3A can edit c.136C>U in *SDHB* RNA *in vitro*, an *SDHB* ORF RNA of ∼1.1 kb with an artificial sequence at its 5′-end was incubated with whole-cell lysates of 293T transfectants. Editing of the RNA at c.136 was quantified by allele-specific RT–PCR with a 5′-primer that was specific to the artificial sequence and using the same 3′-primers as described^16^. Lysate expressing *APOBEC3A* but not a control transfectant induced C>U editing of the exogenous *SDHB* RNA at c.136 in a time- and dose-dependent manner, and this activity was not seen with the heat-inactivated lysate (Fig. 8a). To further validate the RNA-editing activity of APOBEC3A, *in vitro* editing assays were performed with purified APOBEC3A. Incubation of *in vitro*-transcribed *SDHB* RNA with His_6_-tagged APOBEC3A protein showed site-specific editing of the *SDHB* RNA *in vitro* (Fig. 8b). Chelation of zinc in the deamination reaction with *1*,*10*-phenanthroline abolished the editing (Fig. 8b). An ssDNA of 120 bases containing the *SDHB* cDNA sequence (c.37–c.156) too was deaminated at c.136 by the recombinant APOBEC3A protein. However, cytidine deamination of the ssDNA was also observed at other positions (c.117 and c.132); the deaminated residue at both positions occurs in a TC sequence context (Fig. 8c). In contrast, cDNAs of the *in vitro*-synthesized *SDHB* RNA incubated with the APOBEC3A 293T transfectant cell lysates or the pure recombinant enzyme showed no evidence of additional mutations in Sanger sequence analysis of a 619 b segment that spanned exons 1–5 (Fig. 8c). Thus, whereas cytidines of both *SDHB* ssDNA and RNA can be deaminated *in vitro* by APOBEC3A, deamination sites of RNA appear to be highly selective, which may reflect a requirement for a more complex sequence or structure context.

## Discussion

In this study, we demonstrate that APOBEC3A, a CDA highly expressed in myeloid cells, is a C>U RNA-editing enzyme that modifies the monocyte/macrophage transcriptome. The RNA editing in monocytes is activated by hypoxia and IFNs in both independent and additive manners (Figs 1a and 2a), and in monocyte-derived macrophages by M1 but not M2 polarization (Figs 1b and 2a). These findings represent the discovery of the first mammalian RNA-editing CDA enzyme since the identification of APOBEC1 in 1993, unveil a previously unrecognized function for the *APOBEC3* family of genes, markedly expand our knowledge of C>U RNA-editing events and highlight a significant effect of micro-environmental factors on such editing.

The RNA-editing activity of APOBEC3A (Fig. 8b) provides a new perspective to understand the anti-viral and -retrotransposition functions of *APOBEC3A* and possibly other *APOBEC3* genes. APOBEC3A has been shown to strongly inhibit retrotransposons and diverse viruses including parvoviruses, alpharetroviruses, HTLV-1 and HIV-1 in the early stages of infection in myeloid cells^28^,^29^,^30^,^31^. The mechanism by which APOBEC3A inhibits these agents is poorly understood. APOBEC3A-mediated restriction of retrotransposons and adeno-associated viruses occurs with the absence or a rarity of changes to DNA sequences of the restricted agent^25^,^32^,^33^,^34^,^35^,^36^. Replacement of the single murine *APOBEC3* gene with either human *APOBEC3A* or *APOBEC3G* in mouse preserves APOBEC3-mediated restriction of the MMTV and MLV murine retroviruses, but a high level of viral DNA deamination is seen only with the latter^37^. Paradoxically, previous studies also show that CDA active site mutations such as H70R, C101S and C106S markedly diminish or abolish the virus-inhibiting activities of APOBEC3A^25^,^26^. These findings thus imply that the catalytic activity of APOBEC3A that is required for its anti-viral and -retrotransposition function may not necessarily involve DNA deamination. We find that the RNA editing and anti-*LINE-1* retrotransposition abilities of APOBEC3A are similarly affected by E72D, C101S and P134A mutations (Fig. 7c,d). This is consistent with the possibility that the newly discovered RNA-editing activity of the host RNAs by APOBEC3A may provide a DNA deamination-independent mechanism for the inhibition of viruses and retrotransposons by the protein. The association established in this study between upregulation of APOBEC3A-mediated C>U RNA editing of cellular transcripts and hypoxia or IFN treatment of monocytes and M1 polarization of macrophages (Figs 1 and 2a) also supports this notion.

Non-synonymously C>U RNA-edited genes identified in this study may represent factors that mediate the anti-viral and -retrotransposition function of APOBEC3A. Several of the edited genes have already been associated with viral pathogenesis. Examples include *ANKRD17*, which positively regulates viral RNA-sensing RIG-I-like receptor signalling^38^; *EVI2B*, which resides in a region orthologous to a common retroviral integration site in murine myeloid leukemia^39^; *HLA-DMA*, which encodes the A subunit of HLA-DM that catalyses the loading of antigenic peptide into major histocompatibility complex class II molecules^40^; *ITGAX* (*CD11C*) and *ITGB2* (*CD18*), which encode an integrin that is exploited by various viruses for cell entry^41^; *UBE2J1*, which targets major histocompatibility complex class I heavy chains for endoplasmic reticulum-associated degradation, a pathway used by cytomegaloviruses^42^; *VIM* encodes vimentin with which various viruses physically interact for their survival^43^; *XPO1*, which encodes a cargo protein that plays a role in exporting the unspliced *HIV-1* RNA^44^; *AP2A1*, which encodes a subunit of clathrin-associated adaptor complex 2 that is involved in infectious entry of various viruses^45^; and, *SIN3A*, which encodes a transcriptional co-repressor that is incorporated in HIV-1 virions as SIN3A-HDAC1 complex that contributes to efficient reverse transcription in host cells^46^.

APOBEC3A is believed to deaminate foreign but not host genomic DNA in primary cells, and previous studies have demonstrated the deamination activity of the enzyme against ssDNA but not RNA^26^,^47^. Our data (Fig. 2d) suggests that the enzyme deaminates cytidines of RNA within CAUC or its 1-nt. variant motifs that are flanked by palindromic sequences. It thus appears that previous studies failed to observe the RNA-editing activity of APOBEC3A, which is known to bind RNA^26^, in part because they used substrate RNAs containing a nonspecific sequence.

An important finding of this study is that hypoxia independently activates C>U RNA editing to levels comparable to those induced by IFN1 (Figs 1a and 3a). Moreover, stimulation of MEPs by hypoxia and IFN1 together additively increases editing, with levels reaching over 80% for 5 of the 31 genes validated by Sanger sequencing (Fig. 3a). As hypoxia is pervasive in inflamed tissue, this suggests that RNA editing has the potential to substantially alter certain cellular proteins in virus-infected cells *in vivo*. How hypoxia activates C>U RNA editing is currently unknown. Although upregulation of *APOBEC3A* expression may underlie the activation of C>U RNA editing by IFNs^17^ (Figs 1a and 3a), *APOBEC3A* expression in MEPs is downregulated by hypoxia (Fig. 2f). Hypoxic stimulation of C>U RNA editing in these cells may therefore be caused by an alternative mechanism such as enhanced translocation of the enzyme to nucleus, where A>I and APOBEC1-mediated C>U RNA editing are known to occur^5^,^8^. Monocytes routinely encounter hypoxia on their exit from the highly oxygenated bloodstream to inflamed tissues, but the oxygen-sensing mechanisms in these cells are poorly understood^48^. We find that RNAs encoding for both the SDHA and SDHB subunits of mitochondrial complex II are targets of hypoxia-induced C>U editing (Supplementary Data 1), suggesting that suppression of this complex facilitates hypoxia adaptation in pro-inflammatory monocytes and macrophages.

Monocytes and monocyte-derived pro-inflammatory macrophages play an important role in pathogenesis of common diseases including infectious diseases, obesity, cancer, Alzheimer's disease and atherosclerosis^49^. We found that APOBEC3A causes non-synonymous RNA editing of transcripts of the *APP*, *AP2A1*, *CAST*, *LRP10* and *XPO1* genes (Fig. 5c) that are implicated in pathogenesis of Alzheimer's disease through regulation of amyloid precursor protein^50^,^51^,^52^,^53^,^54^. Analyses of RNA sequencing data of this study shows that upregulation of *CD33* gene expression, which is associated with Alzheimer's disease susceptibility^55^, also occurs in MEPs under hypoxia (3.1-fold, false discovery rate (FDR)=0.0002, Fisher's exact test) and in M1 relative to M2 macrophages (2-fold, FDR=0.012, Fisher's exact test). It is thus possible that inflammation and hypoxia create multiple risk factors for chronic diseases such as Alzheimer's disease through RNA editing and altered gene expression in monocytes/macrophages.

In conclusion, our findings reveal an unprecedented extent and level of protein-recoding RNA editing in innate immune cells in response to certain micro-environmental factors associated with inflammation, which is mediated by APOBEC3A. In the light of important role that APOBEC3A plays in restricting diverse viruses and retrotransposons, these findings suggest a deaminase-dependent cellular RNA editing model to further investigate the molecular bases of these restrictions.

## Methods

### Isolation and culture of cells

The TLA-HEK293T 293T human embryonic kidney cell line was obtained from Open Biosystems (Huntsville, AL). PBMCs of anonymous platelet donors were isolated from peripheral blood in Trima Accel leukoreduction system chambers (Terumo BCT, Lakewood, CO) after thrombocytapheresis, in accordance with a protocol approved by the institutional review board of Roswell Park Cancer Institute. A density gradient centrifugation method using polysucrose-containing Lymphocyte Separation Medium (Mediatech, Manassas, VA) was used for PBMC isolation. MEPs were prepared from PBMCs using the well-established cold aggregation method^56^, with slight modification. Briefly, PBMCs were subjected to gentle rocking at 4 °C for an hour and aggregated cells that sedimented through fetal bovine serum (FBS; VWR, Radnor, PA) were collected as MEPs after 0.5–3 h for high monocyte enrichment (∼70% monocytes as assessed by immunofluorocytometry for CD14), or after 8–16 h for mild enrichment (∼20–40% monocytes); the latter was used in all experiments, except for the ones of Fig. 1a. MEPs were cultured at a density of 13–63 × 10^6^ ml^−1^ (mean=29 × 10^6^ ml^−1^, *n*=16 experiments) in 1 or 2 ml per well of 6- or 12-well standard tissue culture plates under 5% CO_2_ in RPMI-1640 medium (Mediatech) with 10% FBS, and 100 U ml^−1^ penicillin and 100 μg ml^−1^ streptomycin (Mediatech). Monocytes and lymphocytes were isolated from MEPs based on light scattering and binding of a phycoerythrin-conjugated mouse anti-CD14 antibody (clone RM052, product number 6699509D, 1:40 dilution, Beckman Coulter, Miami, FL) by flow cytometry on a FACS Aria II instrument with FACS Diva 6.0 software (BD Biosciences, San Jose, CA; Supplementary Fig. 3a). CD14+ monocytes used in the experiment for Fig. 1b were isolated from PBMCs using mouse anti-CD14 antibody-conjugated microbeads and magnetic separation on an AutoMACS instrument (Miltenyi Biotec. Auburn, CA). Monocytes used in the experiment for Fig. 3c were isolated from MEPs by immunomagnetic negative selection using EasySep Human Monocyte Enrichment Kit (Stemcell Technologies, Vancouver, Canada). For the *APOBEC3A* knockdown experiment, monocytes of 70% CD14 positivity were isolated from PBMCs using a centrifugation-based method^57^, with a single layer of iso-osmolar, 42.5% v/v solution of Percoll (GE Healthcare, Pittsburgh, PA) in RPMI-1640 medium with 10% FBS. Except for the cells used in the experiment for Fig. 1b, all primary cells were used in experiments immediately after their isolation from PBMCs. Enhancement by hypoxia of *SDHB* c.136C>U RNA editing was not observed in cultures of previously cryopreserved CD14+ monocytes (Supplementary Fig.7a). Induction of RNA editing was also not consistently observed in hypoxia if freshly isolated MEPs were cultured at a low cell density (<10 × 10^6^ cells per ml (Supplementary Fig.7b).

### Generation and polarization of macrophages

CD14+ monocytes isolated from PBMCs by magnetic sorting and stored frozen in RPMI-1640 with 36% v/v FBS and 10% v/v dimethyl sulfoxide were thawed and cultured for a week at a density of 0.25 × 10^6^ ml^−1^ with 50 ng ml^−1^ recombinant human macrophage colony stimulating factor (Life Technologies, Carlsbad, CA), 1 × GlutaMAX-I (Life Technologies) and 1 mM sodium pyruvate (Mediatech) to generate M0 macrophages. M0 macrophages were also similarly generated from fresh monocytes isolated from PBMCs by the Percoll-based method. For M1 or M2 macrophage polarization, M0 cells were treated for 2 days with 20 ng ml^−1^ recombinant human IFNγ (Life Technologies) and 100 ng ml^−1^ *Escherichia coli* LPS (List Biological Laboratories, Campbell, CA), or 20 ng ml^−1^ recombinant human interleukin 4 (Life Technologies), respectively. RNA was isolated from cells using the Total RNA Purification Kit from Norgen Biotek (Thorold, Canada).

### Hypoxia and IFN treatments

For hypoxia, cells were cultured under 1% O_2_, 5% CO_2_ and 94% N_2_ in an Xvivo System (Biospherix, Lacona, NY). Human IFNγ and ‘universal' type I IFN, a hybrid of amino-terminal IFNα-2 and carboxy-terminal IFNα-1, produced in *E. coli* were obtained from PBL Assay Science (Piscataway, NJ) and used at 200 and 300–1,500 U ml^−1^, respectively. Unless noted otherwise, hypoxia and/or IFN treatments were for 24 h. Differential viability of MEPs after 1-day culture in normoxia versus hypoxia was not observed as evaluated by Trypan blue stain. Transfected 293T cells were subjected to hypoxia and/or IFN treatment 24 h after transfection.

### RNA sequencing of MEPs

Indexed sequencing libraries were generated from RNA, isolated using TRIzol and without DNAse treatment, as per methods and reagents provided with the TruSeq Stranded Total RNA Sample Prep Kit with Ribo-Zero ribosomal RNA reduction chemistry (Illumina, San Diego, CA). PCR for library generation employed ten cycles. Electrophoresis of the libraries on Bioanalyzer 2100 instrument (Agilent, Santa Clara, CA) showed highest peaks at 220–240 bp. Paired-end multiplexed sequencing of libraries (three per flow cell lane) to generate reads of 101 b was performed on HiSeq 2000 instrument with TruSeq SBS and PE Cluster v3 Kits (Illumina). CASAVA 1.8.2 software (Illumina) was used for base-calling and de-multiplexing, to obtain the raw RNA sequencing reads for further analyses. RNA sequencing of all six samples of this study was performed in one batch.

### Macrophage RNA sequencing data of Beyer *et al*

Paired-end, 101-b read sequence data generated using TruSeq RNA Sample Preparation Kit on Illumina HiSeq 2000 for paired M1 and M2 macrophages derived from CD14+ monocytes of three donors^18^ was obtained as SRA files from NCBI SRA (accession number SRP012015). Raw data in fastq format was extracted from the files with fastq-dump utility in NCBI SRA Toolkit 2.3.3 (http://www.ncbi.nlm.nih.gov/sites/books/NBK158900/).

### Processing of RNA sequencing reads

Quality of reads was assessed using FastQC 0.10.1 (www.bioinformatics.babraham.ac.uk/projects/fastqc/). Trimmomatic 0.32 (www.usadellab.org/cms/?page=trimmomatic) was used to trim 12 b from the 5′-end and remove adapter sequences and poor-quality bases from the reads. The Trimmomatic call was invoked with ‘HEADCROP:12 ILLUMINACLIP: TruSeq3-PE-2.fa:2:30:10:6:TRUE LEADING:5 TRAILING:5 SLIDINGWINDOW:4:15 MINLEN:30', to satisfy these criteria, in the following order : (1) remove 12 b from the 5′-end of all reads because of base bias at these positions; (2) remove read segments that matched sequences of adapters and primers used for sequencing library preparation (the TruSeq3-PE-2.fa file provided with Trimmomatic was used); (3) remove leading/trailing bases with Phred_33_ base quality score <5; (4) using a sliding window of four bases, remove the most 5′-base if the average Phred_33_ base quality score of the four bases was <15; and (5) completely discard trimmed reads with <30 remaining bases. Pair-mates of a fraction of raw reads were lost following this read processing with Trimmomatic. Processed read data thus had both paired and unpaired reads (Supplementary Table 1).

### Mapping of processed RNA sequencing read pairs

Processed read pairs were uniquely mapped to the hg19 genome with the Subread^58^ subjunc 1.4.3-p1 aligner. The subread-buildindex command of Subread was used with default argument values to index the whole-genome FASTA file for the UCSC hg19 genome assembly (obtained from Illumina iGenomes). Subread subjunc command was used for mapping paired reads to the genome, using the genome index with arguments u and H, but otherwise default argument values to permit only unique mapping of a read and using Hamming distance to break ties when there were more than one best mappings. The nature of genomic regions that the reads mapped to was assessed using RSeQC 2.3.7 (ref. 59). Mapping statistics are provided in Supplementary Table 2. To obtain gene-level mapped read count data, the mapping results (BAM files) were analysed with Subread featureCounts with reference to the GTF gene annotation file from UCSC (6 March 2013 version) in the Illumina iGenomes UCSC hg19 data, with the following argument values specified: isStrandSpecific (s)=2 (0 in case of the macrophage data), GTF.featureType (t)=exon, GTF.attrType (g)=gene_id, isPairedEnd (p), allowMultiOverlap (O).

### Processed RNA sequencing reads mapping with TopHat2 aligner

Processed reads, both paired and unpaired, were also mapped to the UCSC hg19 human genome assembly with the TopHat2 2.0.10 aligner^60^, permitting only unique mapping of a read with up to three nucleotide mismatches. The bowtie2 index for the UCSC hg19 genome assembly was obtained from ftp.ccb.jhu.edu/pub/data/bowtie2_indexes and the transcriptome index was built with TopHat2 using the GTF gene annotation file from UCSC (6 March 2013 version) in the Illumina iGenomes UCSC hg19 data. The tophat2 command was used with the transcriptome and bowtie2 genome indexes, and the following argument values specified: mate-inner-dist=−50, mate-std-dev=40, max-multihits=1, read-mismatches=3, read-edit-dist=3, no-novel-juncs (and library-type=fr-firststrand, in case of the RNA sequencing data of MEPs). The nature of genomic regions that the reads mapped to was assessed using RSeQC. Mapping statistics are provided in Supplementary Table 3.

### Generation of mapped RNA sequencing read pileups

After clipping overlaps of read pair-mates with clipOverlap utility in bamUtil 1.0.10 (genome.sph.umich.edu/wiki/BamUtil), pileups were produced from the mapping data (BAM files) with mpileup in SAMtools 0.1.19 (www.htslib.org), with computation of base alignment quality disabled (B), ‘anomalous' reads permitted (A), maximum depth (d) set at 80,000, and aligner-reported read mapping quality (Q) >0 and Phred_33_ base quality score (q) >19 required.

### Analyses of mapped RNA sequencing read pileups

Paired comparison of pileups for the three pairs, from three different human donors, of normoxic and hypoxic MEP, or M2 and M1 macrophage samples was performed to identify genome sites with differential RNA editing in MEPs under hypoxia (test samples) compared with normoxia (control samples), or in M1 macrophages (test) compared with M2 macrophages (control). Python 2.7, R 3.0 and shell scripts were used for the analysis. Sites considered for analysis satisfied all of the following criteria regarding the A/T/G/C base-calling reads that covered them: (1) ⩾20 calls (per sample, as for the other criteria here) in both samples of ⩾1 pair and ⩾5 calls in all 6 samples; (2) ⩾50% of calls for the reference human genome base in all test or all control samples; (3) ⩾2 variant (other than the reference base) but identical base calls in ⩾2 test or ⩾2 control samples, with ⩾1 such calls in all test or all control samples, and ≤5 base calls for a different variant in all 6 samples; and, (4) ⩾95% reads with a base call for either the reference or variant nucleotide in all 6 samples (thus, only 1 type of nucleotide change was considered for a site). Variation or editing level for sites was calculated as the ratio of variant base-calling- to the sum of variant and reference base-calling-read counts. Sites were then filtered by editing level, requiring: (1) ⩾2.5% in ⩾2 test or ⩾2 control samples; (2) mean ⩾5% for test or control samples; and (3) range/mean ⩾2 across all 6 samples (to reduce subsequent multiple testing). Variant sites with known maximum population prevalence >20% for identical sequence polymorphism (as per the popfreq_max ANNOVAR database, detailed below), or sites that did not map to a known RefSeq gene (www.ncbi.nlm.nih.gov/refseq), or mapped to either exons of >1 RefSeq genes on both chromosome strands, or mapped to only introns of >1 RefSeq genes on both chromosome strands were excluded. Annotation data (BED files) for RefSeq gene introns and exons for the UCSC hg19 genome assembly were obtained on 21 March 2014, using UCSC Genome Browser (genome.ucsc.edu). The inverted β-binomial (IBB) test for multiple paired count data^19^ was then applied to the remaining variant sites, to identify sites that were differentially edited between the test and control samples. To control false discovery resulting from multiple testing, *q*-values were calculated from IBB test *P*-values using the *q*-value function in the *q*-value Bioconductor package with these argument values specified: pi0.method=bootstrap, robust. Sites that were further considered had *q*-value <0.05 and >2-fold difference in either direction for editing level between test and control samples (fold-change values, capped at an absolute value of 10^4^, were estimated by the IBB test) in analysis of Subread subjunc-aligned RNA-sequencing data, as well as an IBB test *P*-value <0.05 and >2-fold difference in analysis of TopHat2-aligned RNA-sequencing data. Sites were then filtered if either of their 5′-and 3′-, 29-b-long, flanking genomic sequences, respectively, with either the reference or variant base at the 3′-and 5′-end aligned perfectly with the genome at another location; blat 35 (genome.ucsc.edu) was used for this purpose. Finally, for filtering based on sequencing read strand bias, sites were filtered out if in the Subread subjunc-aligned data the variant base was called from a total across all six samples of more than nine forward RNA sequencing reads but no reverse read, or vice versa, or if the number of forward and reverse reads were significantly different for either the test or control samples (IBB test *P*-value <0.05). Numbers of sites that were left after and filtered by different steps of the analysis described here are noted in Supplementary Table 4. RNA-level nucleotide change was deduced from DNA alteration based on the chromosome strand coding for the gene that a site mapped to, using the exon-bearing strand if a site mapped to both an intron and exon on opposite strands.

### Analyses of RNA editing sites

ANNOVAR tool (23 August 2013 release; www.openbioinformatics.org/annovar) and ljb23_metalr (22 February 2014), popfreq_max (21 August 2013), RefSeq-based refGene (13 November 2013) and dbSNP 138-based snp138 (13 December 2013) ANNOVAR databases were used to annotate sites with information such as gene features they are located in, frequencies of known C/T genomic DNA polymorphism and effects on amino acid coding. Coding genomic strand sequences flanking the editing sites were extracted from the whole-genome FASTA file for the UCSC hg19 genome assembly (obtained from Illumina iGenomes) with the getfasta utility in bedtools 2.17.0 (github.com/arq5x/bedtools) and these sequences were analysed as transcript RNA sequences. Palindromic sequence context of editing sites was manually examined. RNA folding was predicted with ViennaRNA package 2.1.6 (www.tbi.univie.ac.at/RNA). These annotations are provided in Supplementary Data 1. Annotations on gene feature and amino acid coding change are summarized in Supplementary Table 5. WebLogo 3 online tool was used to create sequence logos (weblogo.threeplusone.com). Gene-set enrichment analyses for biological function, molecular process and PANTHER pathway ontologies were performed with PANTHER 9.0 (www.pantherdb.org/panther). Enrichment of a gene set with at least two genes for an ontology term, in comparison with the reference database for 21,804 genes, was assessed by binomial test and an FDR <5%, calculated from *P*-values by the Benjamini–Hochberg method, was considered significant.

### Gene expression, RNA and whole-exome sequencing data

Level 3 gene-level expression data determined by RNA sequencing with the UNC v2 pipeline were obtained from Broad Institute GDAC Firehose (2014_03_16 stddata run). RNA and whole-exome sequencing data mapped to the hg19 genome assembly (BAM files) were obtained from Cancer Genomics Hub (University of California, Santa Cruz) during February and March, and October 2014, respectively.

### Analysis of TCGA tumour RNA sequencing data for *SDHB* editing

Pileups were generated as described above. Editing was deemed indeterminable for a sample if <99% of mapped reads had a base call other than A or G, or there were <200 calls with none for A, or there were <100 calls with only one for A (*SDHB* gene is coded on the minus chromosome strand). Otherwise, C>U editing level was estimated as the ratio of G to the sum of A and G calls. Information on C/T single-nucleotide polymorphisms in *SDHB* protein coding sequence was obtained from dbSNP (build 37).

### Differential gene expression analysis of RNA sequencing data

Gene-level raw count values of transcripts were analysed with the edgeR Bioconductor package (version 3.2.3) for normalization with the trimmed mean of *M*-values method and inter-group comparison of gene expression by exact or likelihood ratio tests. For analyses of RNA-sequencing data of tumour samples of TCGA, genes with raw count value >0 for ⩾*N* samples, irrespective of group membership, where *N* equals the size of the *SDHB* c.136C>U editing-positive group, were considered as expressed, and values for prior.df and rowsum.filter parameters in estimateCommonDisp and estimateTagwiseDisp functions of edgeR were set at 0.2 and 4*N*, respectively. An exact test was used to generate *P*-values. For analyses of RNA-sequencing data of MEPs and macrophages, genes with raw count value >1 for ⩾3 samples, irrespective of group membership, were considered as expressed and pair-wise comparison of gene expression between groups using generalized linear models with negative binomial distribution and a likelihood ratio test to generate *P*-values was performed. FDRs were estimated from *P*-values with the Benjamini–Hochberg method and genes with FDR <0.05 were considered as differentially expressed. Summarized results of differential gene expression analyses are provided in Supplementary Tables 7 and 8.

### Gene expression constructs and site-directed mutagenesis

Sequence-verified plasmid constructs in pCMV6 vector for cytomegalovirus (CMV) promoter-driven expression of human *APOBEC3A*, *APOBEC3G*, *CDA* and *SDHB* cDNAs, with sequences matching NCBI RefSeq sequences NM_145699.2, NM_021822.1, NM_001785.1 and NM_003000.2, respectively, for the generation of C-terminal Myc-DDK-tagged APOBEC3A and untagged APOBEC3G, CDA and SDHB proteins were obtained from OriGene (Rockville, MD; product numbers RC220995, SC122916, SC119015 and SC319204, respectively). An inducible bacterial expression construct for APOBEC3A with a C-terminal His_6_-tag in the pET21 vector was obtained from Dr Jinwoo Ahn (University of Pittsburgh, USA). Site-directed mutagenesis of *APOBEC3A* constructs (c.216G>C/p.E72D, c.301T>A/p.C101S or c.400C>G/p.P134A; primer sequences shown in Supplementary Table 10) was performed using Q5 site-directed mutagenesis kit (New England Biolabs, Ipswich, MA). Sequences of cDNA inserts of all of these constructs, except that for *SDHB*, were verified by Sanger sequencing. Insert-less pcDNA 3.1(+) vector (Life Technologies) plasmid was used for control transfectants. The pRL-SV40 plasmid for SV40 promoter-driven expression of Renilla luciferase was obtained from Addgene (Cambridge, MA). A *LINE-1* plasmid^26^ with an ∼6 kb human *LINE-1* element bearing a CMV promoter-driven firefly luciferase cassette in its 3′-untranslated region was obtained from Dr Judith Levin (National Institute of Child Health and Human Development, USA).

### Transfection of plasmid DNA

293T cells were transfected with plasmid DNA using the liposomal X-tremeGENE 9 DNA reagent (Roche, Indianapolis, IN) or jetPRIME (Polyplus-transfection, New York, NY) reagents as per guidelines provided by the reagent manufacturer. Transfection efficiency with both reagents was 60%–80% as assessed by fluorescent microscopy of cells transfected with the pLemiR plasmid DNA (Open Biosystems) for expression of a red fluorescent protein. Cells were harvested 2 days after transfection.

### Knockdown of *APOBEC3A* RNA in M1 macrophages

A day before induction of M1 polarization, M0 macrophages at a density of 1 × 10^6^ cells per ml in 1 ml medium per well of six-well plates were transfected with 100 nM of negative control (Silencer negative control no. 1, product number AM4611, Life Technologies) or either or equimolar mix of two human *APOBEC3A* siRNAs (Silencer 45715 and 45810, respectively, with sense sequences 5′- GACCUACCUGUGCUACGAATT -3′ and 5′- GCAGUAUGCUCCCGAUCAATT -3′, Life Technologies) using Lipofectamine RNAiMAX (Life Technologies) as per guidelines supplied by the manufacturer. IFNγ and LPS were added with 1 ml medium to each well, to induce M1 polarization, and cells were harvested a day later.

### *LINE-1* retrotransposition assay

Briefly, firefly luciferase expression conditional to the retrotransposition of a human *LINE-1* element from a plasmid DNA to the genome is measured in this assay. 293T cells at ∼50% confluence in 12-well tissue culture plates were co-transfected with 0.75 μg of the *LINE-1* plasmid, 0.5 μg of pcDNA 3.1(+) or an *APOBEC3A* expression plasmid, 0.25 μg of pcDNA 3.1(+) and 1 ng of pRL-SV40 plasmid (per well). Transfectants were lysed after 2 days for measurement of their firefly and Renilla luciferase activities using Dual-Luciferase Reporter Assay System (Promega, Madison, WI). Retrotransposition was quantified as the ratio of firefly and Renilla luciferase activities.

### Reverse transcription and PCR

RNA was reverse transcribed with random DNA hexamers and/or oligo-dT primers using material and methods provided with the Transcriptor First Strand cDNA Synthesis (Roche) or High Capacity cDNA Reverse Transcription (Life Technologies) kits. PCR typically employed 35 cycles of amplification and an annealing temperature of 60 °C. PCR oligonucleotide primers (Integrated DNA Technologies, Coralville, IA) are listed in Supplementary Table 9. Electrophoresis of PCR reactions on agarose gel was used to confirm the generation of a single product in a PCR. Primers used for PCR of cDNA templates were designed such that the amplicons spanned multiple exons. A blend of Taq and high-fidelity Deep Vent_R_ DNA polymerases (OneTaq, New England Biolabs) was used in PCR to generate products for Sanger sequencing. For quantitative PCR to assess *ACTB*, *APOBEC3A*, *APOBEC3B*, *CDA*, *SDHB*, *SIN3A* and *B2M* gene expression, reactions using FastStart Taq DNA polymerase and SYBR Green I dye were performed on a LightCycler 480 System (Roche). Quantification cycle (*C*_q_) values were calculated by the instrument software using the maximum second derivative method and the mean *C*_q_ value of duplicate or triplicate PCR reactions was used for analysis. TaqMan Gene Expression Assays from Life Technologies with identification numbers Hs00234140_m1, Hs00171149_m1, Hs00233627_m1 and Hs00267207_m1, or prepared in house^61^ were respectively used to quantify *CCL2*, *CCL19*, *FCER2*, *MRC1* and *ACTB* with PCR performed on a 7900HT instrument (Life Technologies) and *C*_q_ values determined with automatic baseline and threshold detection by SDS 2.4 software (Life Technologies).

### Sanger sequencing

Sequencing primers (Integrated DNA Technologies) are listed in Supplementary Table 9. Candidate C>U RNA-editing sites for which PCR-amplified genomic DNA and cDNA fragments were sequenced are noted in Table 1. PCR reactions were treated with ExoSAP-IT exonuclease (Affymetrix, Santa Clara, CA) and then directly used for sequencing on 3130 xL Genetic Analyzer (Life Technologies). Major and minor chromatogram peak heights at a nucleotide position of interest were quantified with Sequencher 5.0 software (Gene Codes, Ann Arbor, MI), to calculate editing level for the position. As the software identifies a minor peak only if its height is >5% of the major peak's, a relative minor peak height value of 4% was assumed to assign an editing level of 3.8% when a minor peak was absent. Appropriateness of this method to estimate RNA-editing level was confirmed by comparing measurements of *SDHB* c.136C>U RNA-editing level obtained with it against those obtained with allele-specific RT–PCR (Supplementary Fig. 8).

### Immunoblotting of cell lysates

Whole-cell lysates were prepared using M-PER reagent (Thermo Fisher, Rockford, IL) with 1 × Halt protease and phosphatase inhibitor cocktail (Thermo Fisher). Reducing and denaturing polyacrylamide gel electrophoresis of 20 μg proteins in Laemmli buffer system was performed on pre-cast, 4%–15% gradient polyacrylamide gels (Mini-PROTEAN TGX, Bio-Rad, Hercules, CA). Proteins were then transferred to polyvinylidene difluoride membrane with a pore-size of 0.2 μm for 7 min at 1.3 A in a Bio-Rad Trans-Blot Turbo apparatus. Membranes were incubated in Tris-buffered 0.15 M NaCl of pH 7.5 with 0.05% v/v TWEEN 20 (Sigma Aldrich, Saint Louis, MO) and 5% w/v dried, non-fat, cow milk (Carnation, Nestlé, Glendale, CA) with antibodies at dilutions recommended by their manufacturers. Rabbit polyclonal anti-APOBEC3A (product number sc-130688, D-23, 1:200 dilution; used in the experiments for Figs 5a and 7a), anti-APOBEC3A/B (product number sc-292434, H-89; used in the experiment for Fig. 6b, 1:150 dilution), anti-ASCC2 (product number sc-86303, T-16; raised against peptide from internal region of human ASCC2, 1:200 dilution) and anti-TMEM109 (product number sc-133788, D-23; raised against human TMEM109 peptide of undisclosed sequence, 1:200 dilution) antibodies, and mouse monoclonal anti-CDA (product number sc-365292, D-5, 1:500 dilution) and anti-SDHB (product number sc-271548, G-10; raised against human protein of full length, 1:500 dilution) antibodies were obtained from Santa Cruz Biotechnology (Santa Cruz, CA). Rabbit polyclonal anti-APOBEC3G (product number ab38604, 1:8,000 dilution), mouse monoclonal anti-β-actin (product number AM4302, 1:15,000 dilution) and rabbit monoclonal anti-SIN3A (product number MABE607, EPR6780; raised against peptide near C terminus of human SIN3A, 1:3,000 dilution) antibodies were respectively obtained from Abcam (Cambridge, MA), Life Technologies and EMD Millipore (Billerica, MA). Rabbit polyclonal anti-calnexin antibodies (product number GTX10966, C3, 1:2,000 dilution) were purchased from GeneTex (Irvine, CA). Horseradish peroxidase-conjugated, goat anti-mouse or -rabbit IgG antibodies were obtained from Life Technologies and used at 1:2,000 dilution. Luminata Forte Western HRP Substrate (EMD Millipore) and CL-XPosure auto-radiography films (Thermo Fisher) were used for chemiluminescent detection. Used membranes were stripped using a guanidine hydrochloride-based solution for re-probing with a different antibody. Uncropped scans of the immunoblots are shown in Supplementary Fig. 9.

### DNA deamination assay with cell lysates

The deamination assay described by Byeon *et al*.^27^ was used. Whole-cell lysates were prepared using M-PER reagent (Thermo Fisher) with 1 × Halt protease and phosphatase inhibitor cocktail (Thermo Fisher). Briefly, 180 nM 5′ Alexa Fluor 488 fluorescent dye-labelled ssDNA substrate of 40 bases (Integrated DNA Technologies) was incubated at 37 °C for an hour with 10 μl lysate and 10 units of *E. coli* uracil DNA glycosylase (New England Biolabs) in 10 mM Tris (pH 8.0), 50 mM NaCl, 1 mM dithiothreitol (DTT) and 1 mM EDTA in a volume of 50 μl. The reaction was stopped by adding 40 μg proteinase K (Life Technologies) and incubating it for 20 min at 65 °C. Ten microlitres of 1 N NaOH was added to the reaction, which was then incubated at 37 °C for 15 min. After adding 10 μl of 1 N HCl, the reaction (10 μl) was electrophoresed on a 10% denaturing polyacrylamide gel. Typhoon 9400 Imager (GE Healthcare) was used to scan the gel in fluorescence mode.

### Purification of recombinant APOBEC3A protein

Rosetta 2(DE3)pLysS *E. coli* (EMD Millipore) transformed with a bacterial expression construct for *C*-His_6_-tagged APOBEC3A and grown in Luria broth at 37 °C were induced for expression of the recombinant protein with 0.3 mM isopropyl *β*-*D*-*1*-thiogalactopyranoside and cultured overnight at 18 °C. Harvested cells were lysed with a French pressure cell (American Instrument Corporation, Hartland, WI) and Ni-NTA His.Bind Resin (EMD Millipore) was used as per manufacturer's instructions to purify APOBEC3A protein from the lysates by affinity chromatography. Isolated protein was concentrated using an Amicon Ultra-4 Centrifugal Filter Unit with Ultracel-3 membrane (EMD Millipore; nominal molecular weight limit of 3 kDa). The concentrated protein was stored in 25 mM Tris (pH 8.0) with 50 mM NaCl, 1 mM DTT, 5% v/v glycerol and 0.02% w/v sodium azide. Staining with Coomassie blue of protein preparation electrophoresed on a denaturing polyacrylamide gel indicated that it had APOBEC3A at >90% purity.

### *In vitro SDHB* editing assay

Whole-cell lysates of 293T transfectants were prepared using lysis buffer containing 0.2% Surfact-Amps NP-40 (Thermo Fisher), 30 mM *4*-(*2*-hydroxyethyl)-*1*-piperazine-ethane-sulfonic acid (HEPES; pH 7.5), 100 mM KCl, 25 mM NaCl, 1.5 mM MgCl_2_, 1 mM DTT and 0.5x Halt protease and phosphatase inhibitor cocktail, and stored with 10% v/v glycerol at −80 °C. *SDHB* ORF RNA of ∼1.1 kb was generated by *in vitro* transcription of XhoI enzyme-linearized plasmid DNA using reagents and methods provided with the MEGAscript T7 Transcription Kit (Life Technologies). *SDHB* RNA isolated from the transcription reaction was treated with DNAse I (Thermo Fisher) and its integrity verified by electrophoresis on an agarose gel. For *in vitro SDHB* RNA-editing assay, transfectant cell lysate (2–8 μl containing 21–84 μg protein) was incubated at 37 °C for 4–11 h with 50 pg (125 amole) of *SDHB* RNA in a buffer containing 0.02 U μl^−1^ RNAse inhibitor (Protector, Roche), 100 mM KCl, 10 mM HEPES (pH 7.4), 1 mM DTT and 1 mM EDTA in a total volume of 50 μl. *In vitro* assays with purified APOBEC3A contained 5–10 μM APOBEC3A, 50 pg *SDHB* full-length RNA or single-stranded *SDHB* DNA (c.37-c.156), 10 mM Tris (pH 8.0), 50 mM KCl and 10 or 100 nM ZnCl_2_ with or without 10 mM *1*,*10*-phenanthroline (Sigma Aldrich). The reactions were incubated for 2 h at 37 °C. RNA was purified from the reactions containing transfectant lysates or purified APOBEC3A using TRIzol (Life Technologies) as per the manufacturer's instructions. The c.136C>U editing of the exogenous RNA was assessed by allele-specific RT–PCR as described previously^16^ but using a forward PCR primer (5′- GGAATTCGGCACGAGGAC -3′) that does not bind the cDNA of endogenous *SDHB* RNA. For Sanger sequencing to assess a 619-b segment of the RNA that spanned exons 1 to 5, the cDNA was amplified with primers with sequences 5′- GGTCCTCAGTGGATGTAGGC -3′ and 5′- TGGACTGCAGATACTGCTGCT -3′. For reactions with *SDHB* DNA as substrate, 4 μl of the reaction was directly used in PCR of volume 20 μl with primers with sequences 5′- TTGCCGGCCACAACCCTT -3′ and 5′- AGCCTTGTCTGGGTCCCATC -3′ to amplify the substrate for Sanger sequencing by the forward primer.

### Other

*SDHB* gene expression and c.136C>U RNA editing was quantified by RT–PCR^16^. Unless noted otherwise, total RNA, genomic DNA and plasmid DNA were isolated using material and methods provided with TRIzol, DNA Wizard Genomic DNA Purification Kit (Promega) and Plasmid Kit (Qiagen, Germantown, MD), respectively. RNA/DNA was quantified by spectrophotometry on a Nanodrop 2000 instrument (Thermo Fisher). Proteins were quantified using Bio-Rad Dc assay with BSA standards. Statistical tests were two-tailed and were performed using R 3.0, Excel 2010 (Microsoft, Redmond, WA), or Prism 6.0 (GraphPad, San Diego, CA) software.

## Author contributions

B.E.B. designed the study with contributions from S.K.P and S.S. S.S. generated the expression constructs for mutant APOBEC3A and performed the experiments with 293T transfectants and APOBEC3A protein, and. S.K.P. analysed RNA sequencing data and editing sites, and created figures for the manuscript. B.E.B. quantified results of Sanger sequencing and analysed palindromic sequences and RNA folding. S.K.P. and E.D.K. performed the experiments with macrophages. S.M.E. and P.D.G. prepared purified APOBEC3A proteins. R.T.T., B.E.B. and S.S. performed all other experiments. B.E.B., S.K.P. and S.S. wrote the manuscript. S.S. and S.K.P. contributed equally to the study.

## Additional information

**Accession codes:** RNA Sequencing data of MEPs were deposited in NCBI Sequence Read Archive (SRA) with accession number SRP040806.

**How to cite this article:** Sharma, S. *et al*. APOBEC3A cytidine deaminase induces RNA editing in monocytes and macrophages. *Nat. Commun.* 6:6881 doi: 10.1038/ncomms7881 (2015).

## Supplementary Material

Supplementary Figures and Tables Supplementary Figures 1-9 and Supplementary Tables 1-10

Supplementary Data 1 Characteristics of sites identified as differentially RNA-edited under hypoxia or M1 macrophage polarization in analyses of RNA sequencing data

## Figures and Tables

**Table 1:** Candidate sites experimentally examined for validation of C>U RNA editing*.

**Gene**	**Chromosomal position**†	**cDNA and amino acid change**‡	**Editing level (%)**§			
			**MEPs**		**Macrophages**	
			**Normoxia**	**Hypoxia**	**M2**	**M1**
*AP2A1*	19:50295238	C520T, R174X	0	15.5	NA	NA
*APP*	21:27326988	C1546T, R516C	0	8.5	NA	NA
*ASCC2*	22:30221126	C202T, R68X	0.9	19.1	NA	NA
*C1QA*	1:22965523	C361T, R121W	NA	NA	0.1	10.7
*CAST*	5:96106257	C1826T, S609F	0	7.2	0.1	5.2
*CCDC109B*	4:110605624	C638T, S213L	0.3	20.4	NA	NA
*EVI2B*	17:29632509	C119T, S40L	0	18.1	0.6	12.1
*FAM89B*	11:65340979	C437T, P146L	0	16.2	NA	NA
*GLTSCR2*	19:48253494	C349T, R117W	0.5	6.8	0.2	11.2
*GPR160*	3:169801777	C17T, S6L	0.6	15.7	NA	NA
*HLA-DMA*	6:32918428	C241T, R81C	0.6	9.9	NA	NA
*ICAM3*||	19:10444896	C1381T, Q461X	0	18.4	0	7.2
*ITGB2*	21:46319067	C908T, S303L	0.9	5.1	NA	NA
*LGALS9*	17:25967659	C193T, R65W	0.2	7.6	0	5.4
*LRP10*	14:23346296	C1702T, R568X	0	6.2	NA	NA
*NBN*	8:90955531	C2134T, H712Y	4	24.2	0	22.4
*PABPC4*	1:40027426	C1840T, H614Y	4.4	31.7	1.6	39.9
*PCGF3*	4:737366	C367T, R123W	2	22.2	0	12.8
*PPA2*	4:106317458	C319T, Q107X	NA	NA	0.2	8.8
*PRPF40A*	2:153515789	C2404T, R802X	0	5.1	NA	NA
*RGS10*	10:121275109	C311T, S104L	0.2	7.9	0	15.3
*RNH1*	11:499165	C464T, S155L	3	18.5	0	9.2
*SDHB*	1:17371320	C136T, R46X	2.6	23	1.1	15.6
*SIN3A*	15:75668008	C3589T, Q1197X	0	17.2	NA	NA
*SETX*||	9: 135201977	C5008T, Q1670X	1.9	24.6	NA	NA
*SUPT6H*	17:27005584	C1138T, R380X	0.6	12.3	NA	NA
*TMEM109*	11:60687274	C109T, R37X	0	11.2	NA	NA
*TMEM131*	2:98409343	C3650T, S1217L	5.7	26.4	NA	NA
*TMEM179B*	11:62556843	C364T, R122X	NA	NA	0	5.2
*TRAPPC11*	4:184585120	C100T, R34X	0	15.8	NA	NA
*UBE2J1*	6:90048208	C292T, H98Y	4	16.1	1.1	18.9
*VIM*	10:17277300	C1141T, R381C	0.3	15.7	NA	NA
*XPO1*	2:61760990	C43T, Q15X	2.7	10	NA	NA

MEP, monocyte-enriched PBMC; NA, not available.

^*^NA, either editing level was not different between the two groups of samples or it could not be determined

^†^Based on the UCSC hg19 genome assembly used for mapping reads with the Subread subjunc aligner.

^‡^Nucleotide numbering for the shortest transcript isoform, with A of the ATG translation initiation codon at position 1.

^§^Calculated in analysis of RNA sequencing data (Supplementary Data 1); mean value (*n*=3).

^||^Failed Sanger sequencing-based experimental validation.
