# Supplementary material for: APOBEC3A cytidine deaminase induces RNA editing in monocytes and macrophages
Source: Nat Commun. 2015 Apr 21;6:6881. doi: 10.1038/ncomms7881 (PMC4411297; doi:10.1038/ncomms7881)
Supplement: Supplementary Figures and Tables — Supplementary Figures 1-9 and Supplementary Tables 1-10 [file ncomms7881-s1.pdf]

Supplementary Fig. 1

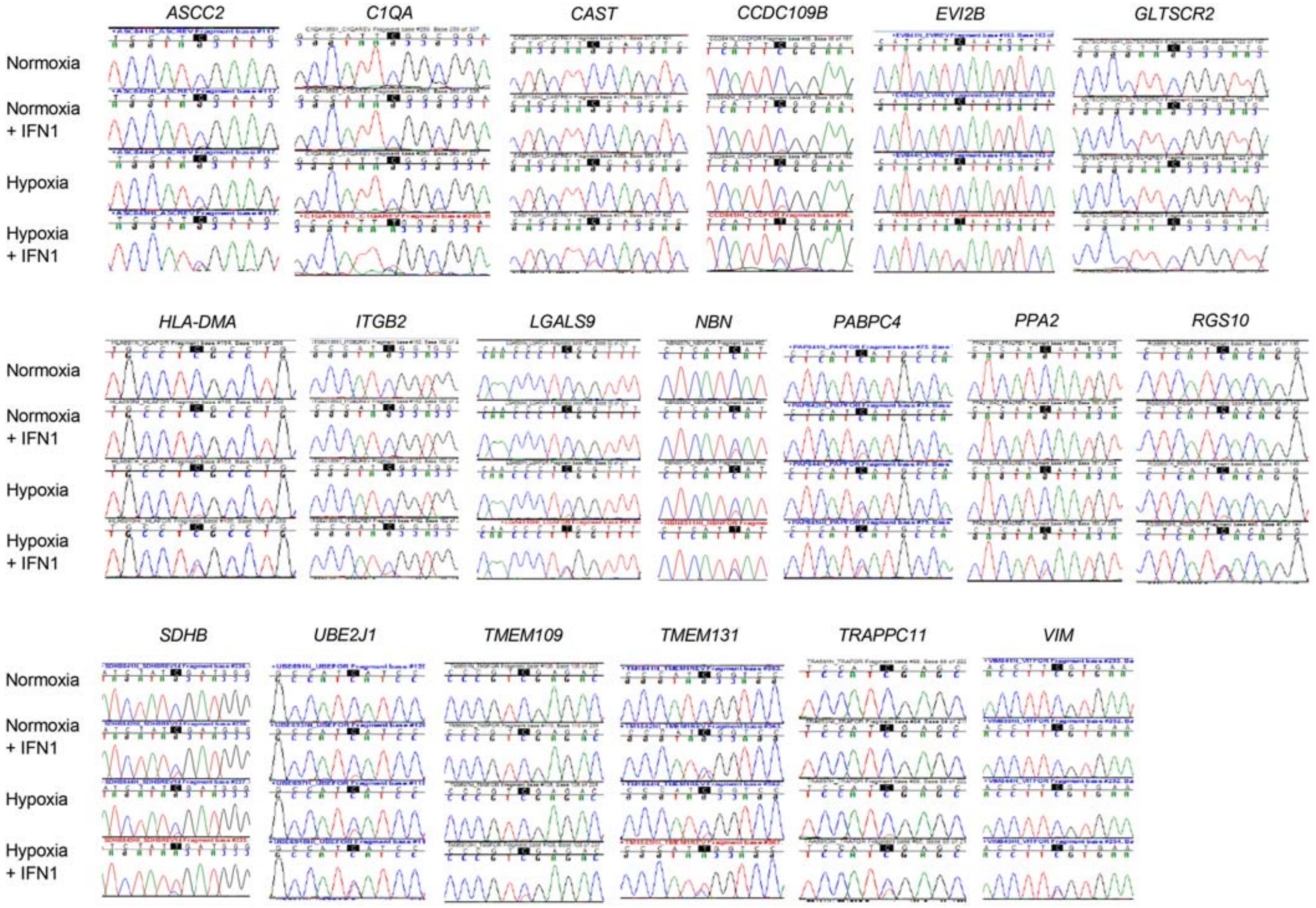

Supplementary Figure 1: *Hypoxia and interferon 1 (IFN1) induce C>U RNA editing in monocyte-enriched peripheral blood mononuclear cells (MEPs)*. Sanger sequence chromatograms of RT-PCR products for 19 genes for which site-specific C>U RNA editing was validated using MEPs of one individual. Cells were optionally treated with hypoxia and/or 600 U per ml IFN1 for 24 hours. Black flags on chromatograms indicate the C>U RNA-edited positions.

Supplementary Fig. 2

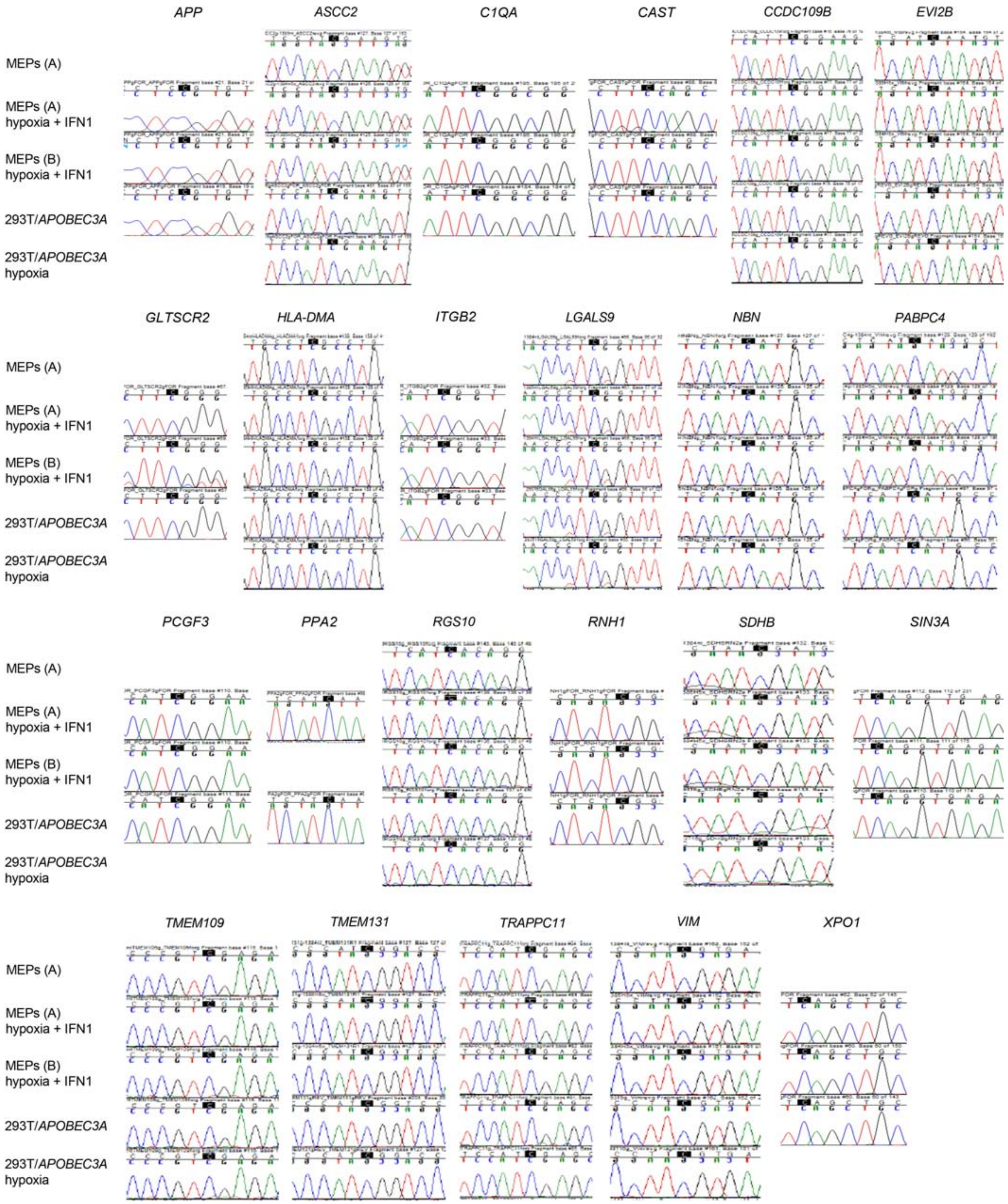

Supplementary Figure 2: *Sanger sequencing of genomic DNA at positions of C>U RNA editing sites.* Genomic DNA of MEPs of two individuals (*A* and *B*) and 293T cells transiently transfected for expression of *APOBEC3A* transfectants, was examined by Sanger sequencing for C>T variation at positions of C>U RNA editing sites for 23 genes. Any C>U RNA editing at the sites was verified by sequencing RT-PCR products of the same cells (Supplementary Figs. 1 and 5). Sequence chromatograms with black flags indicating the C>U RNA-edited positions are shown. MEPs were optionally treated with hypoxia and 600 U per ml IFN1 for 24 hours. The 293T cells were optionally treated with hypoxia for 24 hours one day after transfection. Evidence for C>T variation is not seen in any chromatogram. Some genes were not examined for some of the samples. Some of the data depicted in this figure is also shown in Fig. 5d.

Supplementary Fig. 3

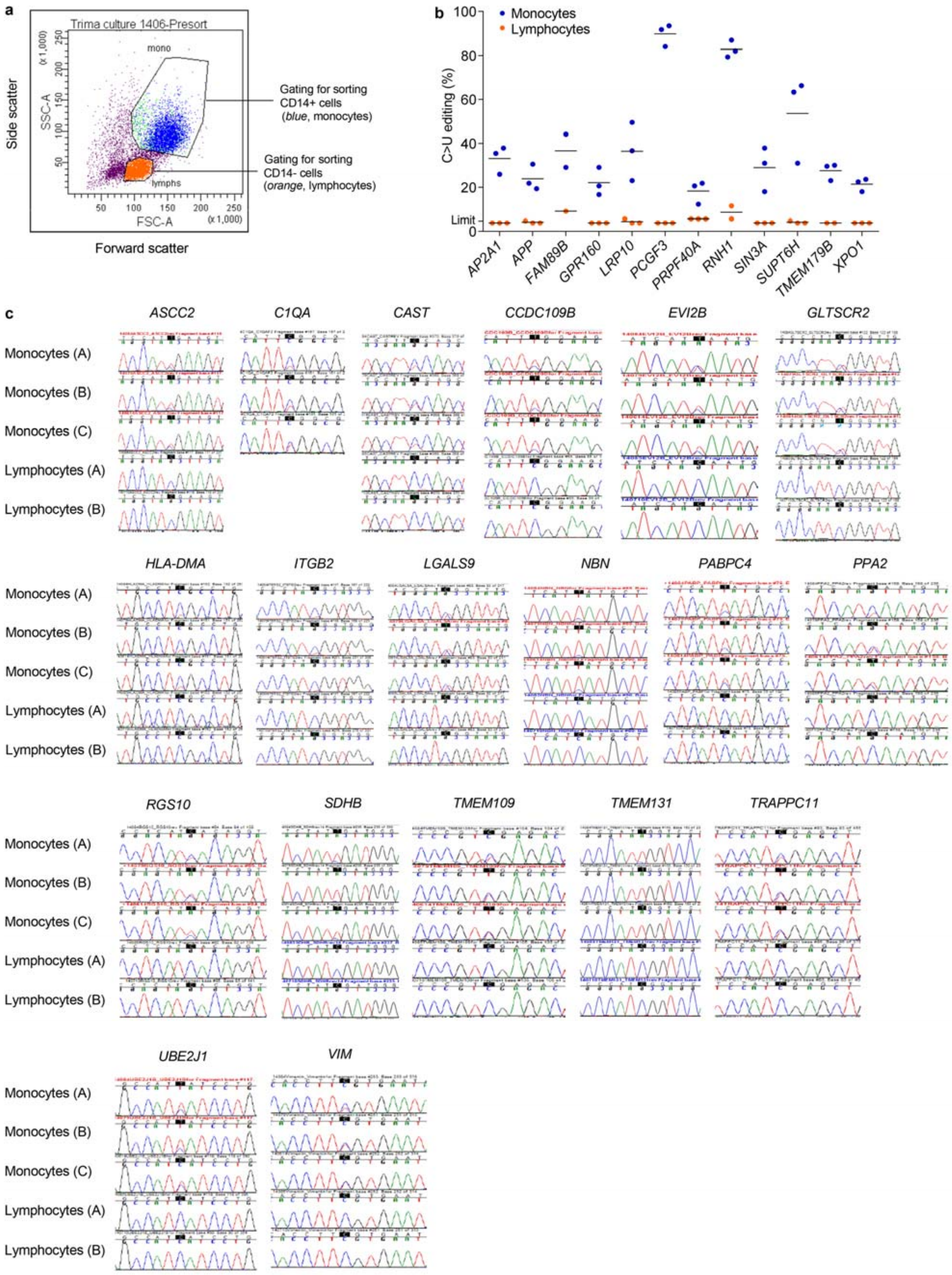

Supplementary Figure 3: *C>U RNA editing by hypoxia and IFN1 in monocytes but not lymphocytes*. (**a**) Dot plot of forward and side scatter values of a sample of MEPs cultured under hypoxia with 600 U per ml IFN1 for 24 hours, indicating the strategy used to isolate monocytes and lymphocytes from MEPs by flow cytometry based on light scattering and cell surface expression of CD14 protein. (**b**) Estimation of site-specific C>U RNA editing by Sanger sequencing of RT-PCR products for 12 genes in monocytes and lymphocytes isolated from hypoxic, IFN1-treated MEPs of three individuals. Editing levels for individual monocyte or lymphocyte samples and their means are shown. A level could not be calculated for *FAM89B* and *RNH1* for some samples because of poor quality of Sanger sequencing. The detection limit for editing (5% level) is indicated. Samples without detectable editing were assigned a level of 3.8%. Information on site-specific C>U RNA editing in the samples for 19 other genes is shown in Fig. 3b. (**c**) Sanger sequence chromatograms of RT-PCR products for another 19 genes for which site-specific C>U RNA editing was validated. Data are shown for monocytes and lymphocytes of three individuals (*A*-*C*). These chromatograms are used to quantify the editing levels that are depicted in Fig. 3b. Black flags on chromatograms indicate the C>U RNA-edited positions. A *C1QA* RT-PCR product for Sanger sequencing could not be obtained for lymphocytes.

Supplementary Fig. 4

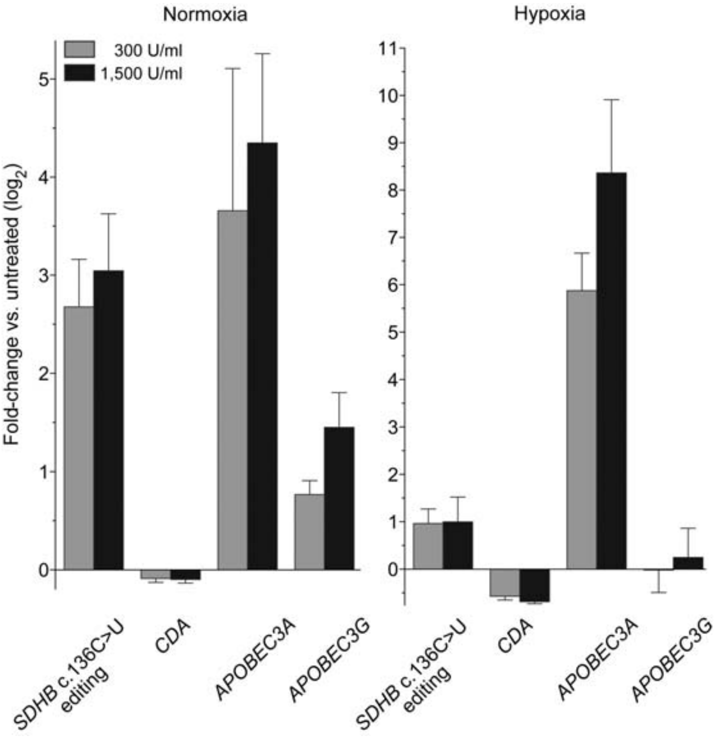

Supplementary Figure 4: *Effect of IFN1 on APOBEC3A, APOBEC3G and CDA gene expression in MEPs*. MEPs of three individuals were optionally treated with 300 or 1,500 U per ml IFN1 for 24 hours under normoxia or hypoxia. *SDHB* c.136C>U RNA editing and expression of *APOBEC3A, APOBEC3G* and *CDA* (normalized to *SDHB*) were quantified by RT-PCR. Mean and range (n = 3) of changes in gene expression and *SDHB* RNA editing relative to untreated cells are shown.

Supplementary Fig. 5

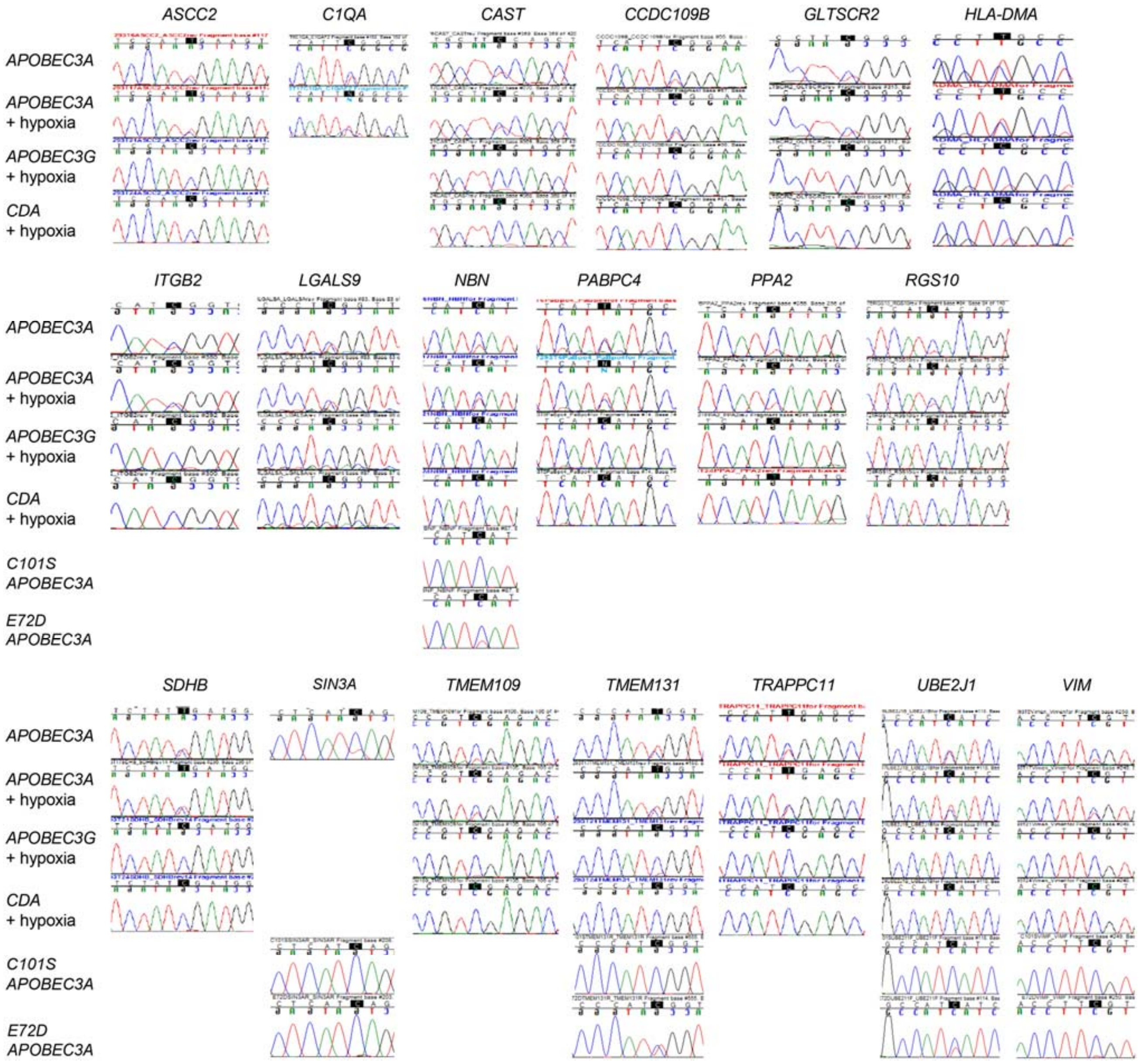

Supplementary Figure 5: *C>U RNA editing in 293T cells transiently transfected for expression of APOBEC3A with or without p.C101S or p.E72D mutation, APOBEC3G or CDA.* Sanger sequence chromatograms of RT-PCR products for 19 genes for which site-specific C>U RNA editing was validated. Cells were optionally treated with hypoxia for 24 hours one day after transfection. Black flags on chromatograms indicate the C>U RNA-edited positions. These chromatograms are used to quantify the editing levels that are depicted in Fig. 6a. Chromatograms of good quality could not be obtained for *C1QA* for hypoxic *APOBEC3A* and *CDA* transfectants. The mutant *APOBEC3A* transfectants were examined for only five genes. Some of the data depicted in this figure is also shown in Fig. 5d.

Supplementary Fig. 6

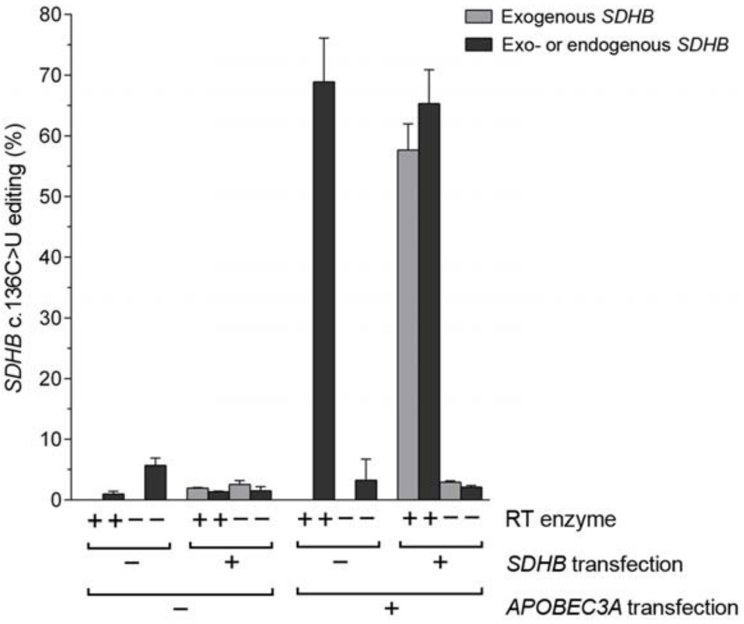

Supplementary Figure 6: *SDHB c.136C>U RNA editing in 293T cells co-transfected with expression constructs for APOBEC3A and SDHB open reading frames*. 293T cells were transiently transfected with the plasmid DNAs. An empty vector was used for cells that did not receive the *APOBEC3A* plasmid. Reverse transcription reactions were performed with or without reverse transcriptase (*RT*), and the products were used as template in allele-specific PCR to quantify editing of either exogenous or both exogenous and endogenous *SDHB* transcripts. Mean and range (n = 3) are shown.

Supplementary Fig. 7

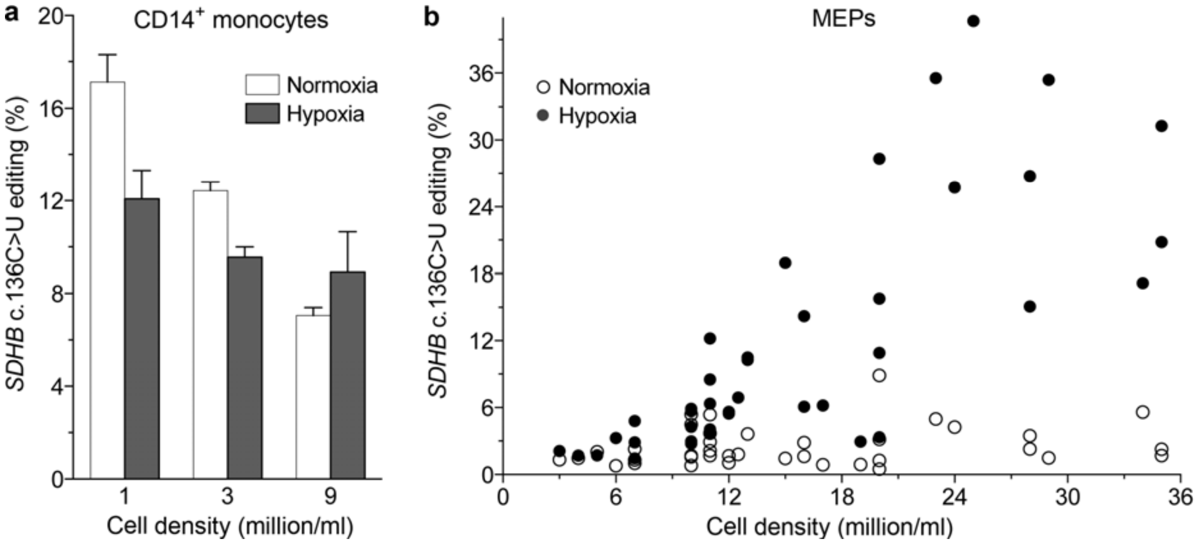

Supplementary Figure 7: *Effect of freeze/thaw and cell density on SDHB c.136C>U RNA editing*. (**a**) CD14^{+} monocytes, isolated from PBMCs of one donor using immunomagnetic beads and stored frozen in RPMI-1640 medium with 36% v/v fetal bovine serum and 10% v/v dimethyl sulfoxide at -80 ºC, were thawed and cultured at indicated density for an hour and then optionally treated with hypoxia for a day. Mean and range (n = 3) of editing levels in normoxic and hypoxic cells are shown. Significant induction of *SDHB* RNA editing by hypoxia (>2-fold, compared to normoxia) is not noticeable. (**b**) In freshly isolated MEPs, a high level of editing under hypoxia was more consistently observed with a cell density above 20 million/ml. The data that is plotted was generated in multiple experiments that had a total of 78 cultures of MEPs isolated from a total of 33 donors. Seventy-six of the cultures were paired; i.e., cells of a specific donor and at a specific density were cultured under either hypoxia or normoxia for a day.

Supplementary Fig. 8

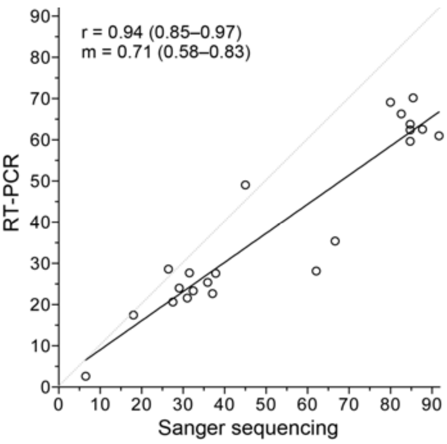

Supplementary Figure 8: *Correlation of SDHB c.136C>U RNA editing level measurements obtained by allele-specific RT-PCR and Sanger sequencing*. The scatterplot shows estimates of editing level determined by both RT-PCR and Sanger sequencing of amplified cDNA for 22 samples of normoxic or hypoxic MEPs. Values of the Pearson correlation coefficient (*r*) and slope (*m*) of the linear regression line (*black*; least squares fitting technique), and their 95% confidence intervals, and the line of identity (*gray*) are also depicted.

Supplementary Fig. 9

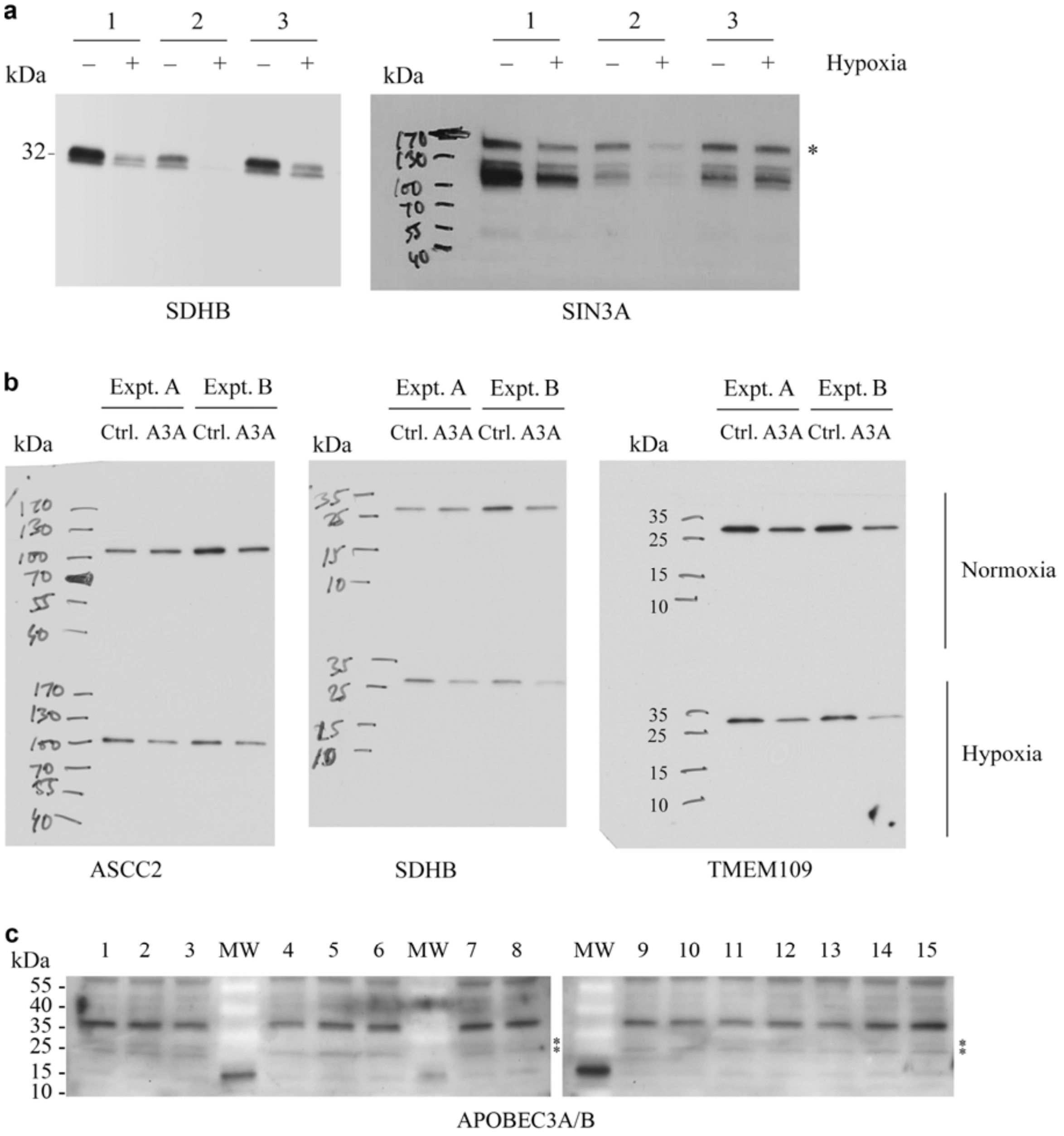

Supplementary Figure 9: *Scans of films of immunoblotting assays whose results are shown in Figs. 3c, 5d and 6b*. (**a**) Cropped views of these scans are shown in Fig. 3c. For SIN3A, that figure shows only the signal at the position marked with an asterisk (*). (**b**) Cropped views of these scans, only for experiment (*Expt.*) *B*, are shown in Fig. 5d. (**c**) Cropped views of these scans are shown in Fig. 6b. That figure shows the two signals at positions marked with an asterisk (*) for lanes that are marked here as 2-3, 5-8 and 13-14. Lanes 1-3, 4-6, 7-9 and 13-15 respectively had protein lysates of the biological triplicates of *Ctrl.*, *1*, *2* and *1 + 2* siRNA transfectants. Molecular weight (MW) markers and the protein(s) being detected are noted in all three panels of the figure.

Supplementary Table 1
Read counts in raw and processed RNA sequencing data

			Processed[b]		*Processing loss (%)*	
Pair[a]	*Condition*	*Raw (read pairs)*	*Read pairs*	*Unpaired reads*	*Read pairs*	*All reads*
			Hypoxia treatment			
1	Normoxia	73,702,071	70,247,604	2,060,835	5	3
1	Hypoxia	61,942,292	58,703,197	1,646,926	5	4
2	Normoxia	72,269,160	68,803,138	1,926,455	5	4
2	Hypoxia	62,143,194	56,856,367	2,103,138	9	7
3	Normoxia	95,646,586	90,824,690	1,693,974	5	4
3	Hypoxia	68,039,772	63,732,067	1,357,476	6	5
			Macrophage polarization			
1	M1	36,443,544	9,598,056	3,402,944	74	69
2	M1	30,952,877	13,434,706	3,392,774	57	51
3	M1	22,072,388	7,201,166	1,219,143	67	65
1	M2	51,417,553	26,546,181	2,442,665	48	46
2	M2	15,585,860	6,367,571	886908	59	56
3	M2	49,258,388	18,315,303	3,698,024	63	59

[a]Cells from three individuals were subjected in a paired design to either normoxia or hypoxia, or M1 or M2 macrophage polarization

[b]Sequencing data after filtering/trimming of reads with Trimmomatic; as per the TopHat2 aligner software

Supplementary Table 2
Mapping of RNA sequencing data with the Subread subjunc aligner

			Mapping rate[c] (%)		*Feature of region that reads mapped to[d] (%)*				
Pair[a]	*Condition*	*Read pairs in input*	*Overall*	*Concordantly paired*	*CDS exon*	*5' UTR exon*	*3' UTR exon*	*Intron*	*Inter-genic*
			Hypoxia treatment						
1	Normoxia	57,775,265	87.7	83.7	18	3	9	60	10
2	Hypoxia	55,020,827	85.3	82.2	16	4	7	62	11
3	Normoxia	61,898,878	85.9	82.0	17	4	7	63	10
1	Hypoxia	69,370,539	89.2	86.4	21	2	9	58	10
2	Normoxia	67,900,180	89.6	84.1	24	3	11	53	10
3	Hypoxia	88,719,827	89.2	84.4	22	3	11	54	11
			Macrophage polarization						
1	M1	7,781,687	95.7	77.9	54	2	27	14	2
2	M1	12,090,174	95.1	80.4	57	3	26	12	2
3	M1	5,897,523	94.6	82.3	53	2	29	13	2
1	M2	23,532,815	95.6	81.2	59	3	26	10	2
2	M2	5,626,748	94.6	82.7	58	3	26	11	3
3	M2	16,016,458	94.3	83.4	56	3	25	14	3

[a]Cells from three individuals were subjected in a paired design to either normoxia or hypoxia, or M1 or M2 macrophage polarization

[b]In sequencing data after filtering/trimming of reads and accepted by the Subread subjunc aligner as input for mapping

[c]As per the aligner software

[d]Distribution over genome of mapped reads was estimated with the RSeQC software. *CDS*, coding sequence; *UTR*, untranslated region; *inter-genic*, within 10 kb up- or down-stream of a transcription start or end site, respectively

Supplementary Table 3
Mapping of RNA sequencing data with the TopHat2 aligner

			Mapping rate[c] (%)		*Feature of region that reads mapped to[d] (%)*				
Pair[a]	*Condition*	*Reads in input[b]*	*Overall*	*Concordantly paired*	*CDS exon*	*5' UTR exon*	*3' UTR exon*	*Intron*	*Inter-genic*
			Hypoxia treatment						
1	Normoxia	142,556,043	87.6	79.3	22	13	9	47	8
2	Hypoxia	139,532,731	86.0	78.0	26	15	11	41	7
3	Normoxia	183,343,354	86.5	77.5	24	17	10	41	8
1	Hypoxia	119,053,320	85.3	76.6	19	20	9	44	8
2	Normoxia	115,815,872	84.2	74.1	17	27	7	43	8
3	Hypoxia	128,821,610	84.6	74.7	17	25	7	44	7
			Macrophage polarization						
1	M1	22,599,056	95.5	73.8	60	3	25	11	2
2	M1	30,262,186	96.4	83.6	63	3	24	9	2
3	M1	15,621,475	96.5	75.0	60	3	26	9	2
1	M2	55,535,027	96.8	82.8	65	3	23	8	2
2	M2	13,622,050	95.9	81.6	63	3	24	8	2
3	M2	40,328,630	96.4	81.3	62	3	23	10	2

[a]Cells from three individuals were subjected in a paired design to either normoxia or hypoxia, or M1 or M2 macrophage polarization

[b]In sequencing data after filtering/trimming of reads and accepted by the TopHat2 aligner as input for mapping

[c]As per the aligner software

[d]Distribution over genome of mapped reads was estimated with the RSeQC software. *CDS*, coding sequence; *UTR*, untranslated region; *inter-genic*, within 10 kb up- or down-stream of a transcription start or end site, respectively

Supplementary Table 4
Number of candidate sites along different steps of analysis of pileups of Subread-aligned RNA sequencing reads for identification of differentially RNA-edited sites[a]

	Left after the step		*Removed by the step*	
Filtering criteria used at step	*MEPs*	*Macrophages*	*MEPs*	*Macrophages*
Adequate (a) read coverage, (b) number of variant-calling sequencing reads, and (c) editing level	54,000	5,058	millions	millions
<20% maximum population prevalence of genomic sequence variation (polymorphism)	51,290	4,419	2,710	639
Location in a known RefSeq gene	31,906	4,099	19,384	320
If location in exons of multiple genes, or in only introns of multiple genes, then all genes coded on the same chromosome strand	31,548	4,063	358	36
q-value <0.05 in test vs. control comparison of editing level	8,578	276	22,970	3,787
Fold-change >2 in test vs. control comparison of editing level	8,458	275	120	1
All of above satisfied with TopHat2-aligned sequencing data[b]	3,750	153	4,708	122
Unique sequence (blat query of genome)	3,184	142	566	11
Insignificant sequencing read strand bias of variant base-calls	3,166	141	18	1

[a]Separate analyses of RNA sequencing data of MEPs and macrophages

[b]Except for raw inverted beta-binomial test P <0.05 instead of q-value <0.05

Supplementary Table 5

Summary of Supplementary Data 1 for genomic feature and effect on translation codon of RNA editing at positions for which the editing level was differentially affected by hypoxia or macrophage polarization[a]

			Hypoxia treatment			*Macrophage polarization*		
		Base alteration:	*A>G*	*C>U*	*Other*	*A>G*	*C>U*	*Other*
5' UTR			3	15	0	0	3	0
Exonic	Synonymous		12	73	3	0	66	0
Exonic	Non-synonymous	Nonsense	0	14	1	0	6	0
Exonic	Non-synonymous	Stop loss	0	0	0	0	0	0
Exonic	Non-synonymous	Missense	18	41	1	1	21	2
Exonic	Non-synonymous	Unknown	0	1	0	0	2	0
Exonic	Non-coding RNA		22	1	1	0	0	0
3' UTR	Coding RNA		164	42	17	14	24	2
3' UTR	Non-coding RNA		11	1	0	0	0	0
Intronic	Coding RNA		2,568	20	28	0	0	0
Intronic	Non-coding RNA		91	3	12	0	0	0
Splicing			0	0	1	0	0	0
Intergenic			2	0	0	0	0	0
		Total:	2,891	211	64	15	122	4

[a]As annotated by ANNOVAR

Supplementary Table 6

Enrichment for ontologies of genes for sites with C>U RNA editing differentially affected by hypoxia or M1 macrophage polarization[a]

Ontology term	*Genes in reference database (n = 21,804)*	*RNA-edited genes*		*P*[b]	*FDR*[c]
		Observed	*Expected*		
Hypoxia treatment (n = 199 genes, 211 sites)					
GO Biological Process					
None					
GO Molecular Function					
Binding	5,933	72	52.8	1.66E-03	2.51E-02
Catalytic activity	5,529	68	49.2	1.70E-03	2.51E-02
Protein binding	2,855	41	25.4	1.26E-03	2.51E-02
Hydrolase activity	2,332	36	20.8	7.45E-04	2.51E-02
GTPase activity	254	8	2.3	2.16E-03	2.55E-02
PANTHER Pathway					
Integrin signalling pathway	175	11	1.6	6.38E-07	1.34E-05
Huntington disease	142	9	1.3	6.35E-06	6.67E-05
Parkinson disease	88	5	0.8	1.24E-03	8.68E-03
Alzheimer disease-presenilin pathway	109	5	1.0	3.11E-03	1.63E-02
Macrophage polarization (n = 116 genes, 122 sites)					
GO Biological Process					
Protein metabolic process	2,807	31	14.9	4.89E-05	3.23E-03
Metabolic process	8,613	63	45.8	8.76E-04	2.15E-02
Antigen processing and presentation	81	4	0.4	9.79E-04	2.15E-02
GO Molecular Function					
Catalytic activity	5,529	46	29.4	5.04E-04	1.76E-02
Hydrolase activity	2,332	23	12.4	2.60E-03	3.03E-02
GTPase activity	254	6	1.4	2.49E-03	3.03E-02
Structural molecule activity	1,261	14	6.7	7.25E-03	3.63E-02
Peptidase activity	747	10	4.0	6.72E-03	3.63E-02
Structural constituent of ribosome	204	5	1.1	4.88E-03	3.63E-02
Cysteine-type endopeptidase inhibitor activity	21	2	0.1	5.75E-03	3.63E-02
PANTHER Pathway					
Integrin signalling pathway	175	8	0.9	5.07E-06	8.62E-05
Parkinson disease	88	5	0.5	1.18E-04	1.00E-03
Huntington disease	142	5	0.8	1.03E-03	5.84E-03
Alzheimer disease-presenilin pathway	109	4	0.6	2.87E-03	1.22E-02
Inflammation mediated by chemokine and cytokine signaling pathway	233	5	1.2	8.41E-03	1.79E-02
Ras Pathway	73	3	0.4	7.18E-03	1.79E-02
Glycolysis	21	2	0.1	5.75E-03	1.79E-02
Axon guidance mediated by Slit/Robo	25	2	0.1	8.04E-03	1.79E-02
Cytoskeletal regulation by Rho GTPase	82	3	0.4	9.83E-03	1.86E-02
FGF signaling pathway	115	3	0.6	2.39E-02	3.76E-02
Blood coagulation	45	2	0.2	2.43E-02	3.76E-02
EGF receptor signaling pathway	123	3	0.7	2.84E-02	4.02E-02

[a]Gene set enrichment analyses, for sets with at least two genes, were performed with PANTHER 9.0

[b]Binomial test

[c]False discovery rate estimated from P values with the Benjamini-Hochberg method

Supplementary Table 7

Genes differentially expressed between tumor samples of the Cancer Genome Atlas (TCGA) that are positive positive or negative for *SDHB* c.136C>U editing in all three cancers[a]

		Log_2 fold-change[b]		
Symbol	*ID*	*HNSC*	*LUAD*	*SKCM*
APOBEC3A	200315	1.49	1.43	2.84
GNLY	10578	1.15	2.19	2.29
CHIT1	1118	1.36	0.98	2.96
ADAMDEC1	27299	1.06	0.80	1.84
LGALS2	3957	0.76	0.92	2.02
TBX21	30009	0.64	1.38	1.57
CLEC4E	26253	0.69	0.79	1.97
PLA2G7	7941	0.74	0.81	1.84
CXCL11	6373	1.10	0.95	1.32
LYZ	4069	0.61	1.03	1.74
LILRB4	11006	0.98	0.66	1.51
IL18RAP	8807	0.51	0.57	1.99
MYO1G	64005	0.56	0.95	1.56
RSAD2	91543	0.84	0.73	1.48
FPR2	2358	0.85	0.70	1.43
SIGLEC1	6614	0.86	0.71	1.39
HK3	3101	1.02	0.58	1.30
CMPK2	129607	0.71	0.59	1.54
LILRB5	10990	0.65	0.71	1.41
ISG15	9636	0.98	0.70	1.08
FGR	2268	0.57	0.59	1.53
LILRA6	79168	0.81	0.53	1.34
NCF1B	654816	0.65	0.54	1.47
SLC15A3	51296	0.68	0.59	1.37
NCF1C	654817	0.62	0.69	1.29
NCF1	653361	0.62	0.58	1.39
OSCAR	126014	0.72	0.60	1.24
IGSF6	10261	0.60	0.56	1.37
MX1	4599	0.59	0.72	1.21
LILRB3	11025	0.74	0.60	1.16
CXCR3	2833	0.66	0.53	1.26
NFAM1	150372	0.61	0.52	1.30
SIGLEC5	8778	0.57	0.58	1.18
ITGAX	3687	0.69	0.61	0.95
SIGLEC14	100049587	0.52	0.65	1.06
IFITM1	8519	0.59	0.51	1.04
TMEM20	159371	-0.98	-0.54	-0.72

[a]Only genes with absolute log_2 fold-change >0.5 were considered; genes are ordered by decreasing mean of the three log_2 fold-change values

[b]*SDHB* c.136C>U editing- positive vs. -negative tumors

Supplementary Table 8

Differential expression of genes coding for known RNA editing and cytidine deaminase enzymes following hypoxia treatment of MEPs, M1 (vs. M2) macrophage polarization, or between *SDHB* c.136C>U editing-positive and -negative TCGA tumor samples[a]

			TCGA tumor samples		
Gene	*Hypoxia treatment (MEPs)*	*Macrophage polarization*	*HNSC*	*LUAD*	*SKCM*
ADAR	ns	ns	NE	NE	NE
ADARB1	0.99	1.14	ns	ns	ns
ADARB2	ns	NE	NE	NE	NE
AICDA	NE	NE	NE	NE	ns
APOBEC1	NE	NE	NE	NE	NE
APOBEC2	NE	NE	ns	NE	NE
APOBEC3A	-1.88	6.24	1.49	1.43	2.84
APOBEC3B	-2.18	2.46	0.40	ns	ns
APOBEC3C	-0.88	ns	0.30	ns	ns
APOBEC3D	-0.71	1.40	0.55	ns	0.88
APOBEC3F	-1.18	ns	0.37	ns	ns
APOBEC3G	-1.45	1.55	0.54	ns	ns
APOBEC3H	ns	NE	NE	ns	1.19
CDA	3.38	1.72	ns	ns	1.84

[a]Log_2 fold-change expression values are shown for genes identified as differentially expressed in hypoxic vs. normoxic MEP, M1 vs. M2 macrophage, or *SDHB* c.136C>U editing-positive vs. -negative cancer tumor tissue comparisons; *ns*, statistically insignificant for differential expression (FDR ≥0.05; see *Methods*); *NE*, identified as not expressed (see *Methods*)

Supplementary Table 9
Sequences of DNA oligonucleotides used as PCR or sequencing[a] primers

Gene	*Forward primer*	*Reverse primer*	*Genomic span (bp)*	*Amplicon size (bp)*
		cDNA template		
ACTB	AGCCTCGCCTTTGCCGA	CTGGTGCCTGGGGCG	981	174
AP2A1	TGACATCCCCCGCATCCTGG	CATGTGCTGGTCATTGAGCAG*	9,367	157
APOBEC3A	CACACATATTCACTTCCAACTTTAAC	GTCCAGGCGCTCCACTTC	80	80
APOBEC3G	GGCCGAGGACCCGAAGG	TTCTGACACAGGCTGCGAAG	99	99
APP	ATGTCCGCGCAGAACAGAA	CAACTTCATCCTGAATCTCCT*	1,135	198
ASCC2	CTTTGACGAGACTCTACAGAAG	AAAGAGCACGCAGAGGTCCAG*	2,881	259
C1QA[b]	AGGCAGGAAGACCTGGCAGA	GGGTACAGTGCAGACGAATC*	1,418	365
CAST	AAACCTGCAGATGACCAAGAC	AGTCATCTTTTGGCTTGGAAG*	3,771	199
CCDC109B	AGAGAGAGCACCATTTACTG*	ATATCCCAGGAGTACACCCA	1,893	193
CDA	CTCACCCAGGAGGGGAGAATC	CGATAGCAATTGCCCTGAAATCC	15,780	257
EVI2B	GCAAAAGAAGTTAACAGCTGAG	GTTGGTTGTCCAGCAGTGACT*	8,636	288
FAM89B	TCAACCTCGACTCAGCGCTG	GATGTGGAAGGCATCCTGCA*	713	321
GLTSCR2	TTGTTGTCAGAGGCCCCA	TCCTTCCGCCTGAGCTTCTT*	3,985	218
GPR160	GGAAGATCATCAGTCAAGGAAG	CCCAAGTATGATCAAGAATAGC*	4,211	204
HLA-DMA	TGGCTGCTACCCCACTCCTG*	GCCCTATTTGCTGGATCATCC	2,431	286
ITGB2	AATCGGCTGGCGCAACGTCA	CTCGTAGGTCTTCACCATCC*	1,404	247
LGALS9	CTCAGCTCCAGTGGAACCAG	AGGATCCCGTTCACCATCAC*	3,600	242
LRP10	CACCTGCAAGCTCTATGCCAT*	CAAGGGGAGCAGCAGAAGG	820	383
NBN	AAATCCATCTGGCATAAATGATGA*	CTCCATTTCCTGCCTTAGCC	2,948	175
PABPC4	GGGAATGCTGCTGGAGATAG	TCAAACCTTGAGTTGAATTCCATA*	1,070	223
PCGF3	TGGTACCAGGCCTCCAAG	CTGCGGTGGTAGTCGTTGTC*	7,177	211
PPA2	GATGATGTTAAGAAGTTCAAACC	ATCTTGCTTCCTCTTGAGTGCA*	12,579	265
PRPF40A	GAGGACATAACTCTAGAATCTG	GATTTATGTCTCCTCTTCTTAC*	3,717	308
RGS10	GAAAATGCAAGATAAGACGCAG	CTGGTCCTGGAGTTTCTGGA*	10,545	166
RNH1	GCTGCACCTCAGCGACAAC	CTGAGCCTGGAGCTGGGGT*	1,150	426
SDHB	GGTCCTCAGTGGATGTAGGC	GTCAAAGTAGAGTCAACTTCAT*	21,036	362
SIN3A	TGTGGATAGTCTGGATAAGCT	GCACATGCTCCTTGGTCCA*	3,649	194
SUPT6H	TGATTACCTAGACCGAGGGC	ATGCCATCAGCAAGAGGTTTGT*	850	312
TMEM109	ATAGCAGTGGCCTGAGAAAG*	CTCTGACACCAGGTGCATGG	5,682	265
TMEM131	ATCTCAGGAGAATCGTTGGT	AGCAGACTGTGAAGAAGCTGCT*	673	263
TMEM179B	GGCGATGACTCGGACCCAG*	CCAAATTCACCCAAGAAGAG	2,399	443
TRAPPC11	TGTGACATCGTAAACATGAG*	GGAATGTACCACTCATATGAAG	2,430	245
UBE2J1	CCAGACTCCGATTTTGATGGAG*	GCTCTTCTTTCCTCAGGAGTG	4,200	272
VIM	GCAGAAGAATGGTACAAATCCA*	CTGTAGGTGGCAATCTCAATG	1,455	344
XPO1	GCATCAGCAAGAGCAACAG*	TCAGTACTTCTTGAGCCATTC*	11,707	249
		Genomic DNA template		
ASCC2	CTTTGACGAGACTCTACAGAAG*	GTCATGGGATCAGTGGCTTAC*	187	187

CCDC109B	GCAAGAGTTCCTTTTCATCTTGC*	ATATCCCAGGAGTACACCCA	212	212
EVI2B	CTTTCATTATAAACCCGCTATAG	GTTGGTTGTCCAGCAGTGACT*	289	289
HLA-DMA	TCACCCTGTGTCCTTTGCAG*	GCCCTATTTGCTGGATCATCC	272	272
LGALS9	GAAGACTATTTGCTTTCCCTGG*	GGAGGGGATTCTTGCTCAC	249	249
NBN	TATGCATGATTTACCATCTTTGC*	CTCCATTTCCTGCCTTAGCC	212	212
PABPC4	GTTGGCTTTGGTCCACCAC*	TGAAAATAGCCACACATACGG*	211	211
RGS10	CTTTCTCTTGCCACAGCAGCT*	CTGGTCCTGGAGTTTCTGGA	253	253
SDHB	CCAGCAAAATGGAATTATCTTGT	CTCTCCTTCAATAGCTGGCTT*	233	233
TMEM109	GCACTCAGCTCACTGTGCTT*	CTCTGACACCAGGTGCATGG	277	277
TMEM131	CAGTTGGTCCCTTTTTCAGC	AGCAGACTGTGAAGAAGCTGCT*	245	245
TRAPPC11	TGTGACATCGTAAACATGAG*	AACACATGCCATCACAATGCC	245	245
UBE2J1	TGCATTGTTTATTCCTCAGGC*	GCTTTGTAAAAGCAACTGGGT*	220	220
VIM	CACCAGACATCTTTCTCACC	ACGCATCCAAGTCTGAGTTCC*	274	274

[a]Oligonucleotide used as a sequencing primer is indicated with an asterisk

[b]Also sequenced with oligonucleotide with sequence GCATCCGGACAGGCATCCAA

Supplementary Table 10

Sequences of DNA oligonucleotides used for site-directed mutagenesis of *APOBEC3A* coding sequence[a]

Mutation	*Forward primer*[b]	*Reverse primer*
c.216G>C/p.E72D	GCCATGCGGAcCTGCGCTTCT	GGCCGTAAAAGCCACAGAG
c.301T>A/p.C101S	CTGGAGCCCCaGCTTCTCCTG	GAGATGAACCAAGTGACCCTG
c.400C>G/p.P134A	TGATTACGACgCCCTATATAAGGAGG	TAGATGCGGGCAGCGAAG

[a]Reference sequence is NCBI RefSeq NM_145699.2

[b]Nucleotide corresponding to the mutation is lowercased
